# Cancer Grade Model: a multi-gene machine learning-based risk classification for improving prognosis in breast cancer

**DOI:** 10.1038/s41416-021-01455-1

**Published:** 2021-06-15

**Authors:** E. Amiri Souri, A. Chenoweth, A. Cheung, S. N. Karagiannis, S. Tsoka

**Affiliations:** 1 Department of Informatics, Faculty of Natural and Mathematical Sciences, King’s College London, London, UK; 2 St. John’s Institute of Dermatology, School of Basic and Medical Biosciences, King’s College London, London, UK; 3 NIHR Biomedical Research Centre at Guy’s and St. Thomas’ Hospitals and King’s College London, London, UK; 4 Breast Cancer Now Research Unit, School of Cancer and Pharmaceutical Sciences, Guy’s Cancer Centres, King’s College London, London, UK

**Keywords:** Machine learning, Breast cancer, Predictive markers, Data integration

## Abstract

**Background:**

Prognostic stratification of breast cancers remains a challenge to improve clinical decision making. We employ machine learning on breast cancer transcriptomics from multiple studies to link the expression of specific genes to histological grade and classify tumours into a more or less aggressive prognostic type.

**Materials and methods:**

Microarray data of 5031 untreated breast tumours spanning 33 published datasets and corresponding clinical data were integrated. A machine learning model based on gradient boosted trees was trained on histological grade-1 and grade-3 samples. The resulting predictive model (Cancer Grade Model, CGM) was applied on samples of grade-2 and unknown-grade (3029) for prognostic risk classification.

**Results:**

A 70-gene signature for assessing clinical risk was identified and was shown to be 90% accurate when tested on known histological-grade samples. The predictive framework was validated through survival analysis and showed robust prognostic performance. CGM was cross-referenced with existing genomic tests and demonstrated the competitive predictive power of tumour risk.

**Conclusions:**

CGM is able to classify tumours into better-defined prognostic categories without employing information on tumour size, stage, or subgroups. The model offers means to improve prognosis and support the clinical decision and precision treatments, thereby potentially contributing to preventing underdiagnosis of high-risk tumours and minimising over-treatment of low-risk disease.

## Background

Despite progress in early detection and personalised targeted therapy, breast cancer remains a major cause of fatality and quality of life reduction worldwide [1]. As breast cancer encompasses a heterogeneous group of diseases, precision diagnosis and treatment mandates accurate tumour stratification into clinically distinct subgroups [2]. Classifying tumours based on intrinsic features like histological grade or subtype can predict disease behaviour more accurately than time-dependent prognostic factors such as tumour size and stage [1, 3, 4], and thereby provide better insight into prognosis and suitable treatment strategies [5, 6].

A significant advance has been achieved in determining treatment on the basis of subtypes of breast cancer, which are immunohistochemically classified based on the expression of oestrogen receptor (ER), progesterone receptor (PR) and human epidermal growth factor receptor 2 (HER2) [7, 8]. ER-positive cancers can benefit from hormone therapies such as tamoxifen or aromatase inhibitors, while the monoclonal antibody trastuzumab is typically used as an adjuvant therapy for HER2 breast cancer in combination with chemotherapy. Patients with triple-negative breast cancer (TNBC), whose tumours lack ER, PR and HER2 expression, do not benefit from the current development of targeted therapies, with treatment options mostly relying on primary surgery, radiotherapy and chemotherapy cocktail [9]. Four further molecular subtypes (luminal A, luminal B, HER2-enriched and basal-like) are identified by PAM50 classification [10] based on the expression of 50-gene signatures, with most basal-like carcinomas, which are usually triple negative, having aggressive phenotype and high relapse rates. Despite significant progress in understanding disease subtypes, the challenge of matching patient clinical characteristics and tissue molecular patterns to prognosis or to a therapeutic strategy remains pertinent [11, 12]. Predictive models of molecular profiling are urgently needed to prevent the underdiagnosis of high-risk tumours and to minimise the over-treatment of low-risk disease, which may help reduce the need for aggressive systemic therapies [13, 14].

Histological grade is a well-described prognostic factor, reflecting tumour morphological characteristics and clinical behaviour of the disease [4, 5]. For instance, in a process of systematic treatment selection, prompt consideration of neoadjuvant or adjuvant chemotherapy is needed for grade-3 tumours, while patients with grade 1 could benefit from long-term follow-up [15]. However, for 30–60% of breast tumours diagnosed as grade 2, treatment may be difficult to assign, as they represent an intermediary and highly variable state in morphology, underlying biology and risk of distant metastasis recurrence [4]. Therefore, patients diagnosed with these tumours are at risk of under-treatment or over-treatment [14]. It has been suggested that only grades 1 and 3 be used towards treatment choice [16] and that grade 2 are not informative [3] without additional metrics [15]. Therefore, accurate stratification of grade-2 tissues poses significant challenges.

The key to resolving these challenges is the development of integrative, systems-level analyses that can capture the multiple facets of disease while also guiding the search for specific molecular cascades that discriminate between disease phenotypes [17]. Analysis of high-throughput gene expression [18] profiles in cancer tissues through computational methods with predictive capabilities [19, 20], such as machine learning models, is critical [13, 21]. Here, we report a bioinformatics strategy where transcriptomic profiles across multiple datasets were integrated and a machine learning model was generated to classify tumours into relevant histological grades. The resulting Cancer Grade Model (CGM) was then used to dissect the molecular subtypes present in grade-2 and unknown-grade cancers and re-classify them into grade-1-like (low-risk) or grade-3-like (high-risk) categories. By interpreting the classification model, key genes were extracted to predict metastasis, risk of relapse and overall survival (OS), regardless of traditional histologically defined receptor status. These markers might also provide potential therapeutic targets for the disease currently lacking treatment options. We report the application of gradient boosted trees on a large dataset of samples integrated from multiple breast cancer studies; however, it is important to note that this strategy can be applied to other types of high-throughput data or cancer types in the future.

## Materials and methods

### Computational framework

**Fig. 1:**
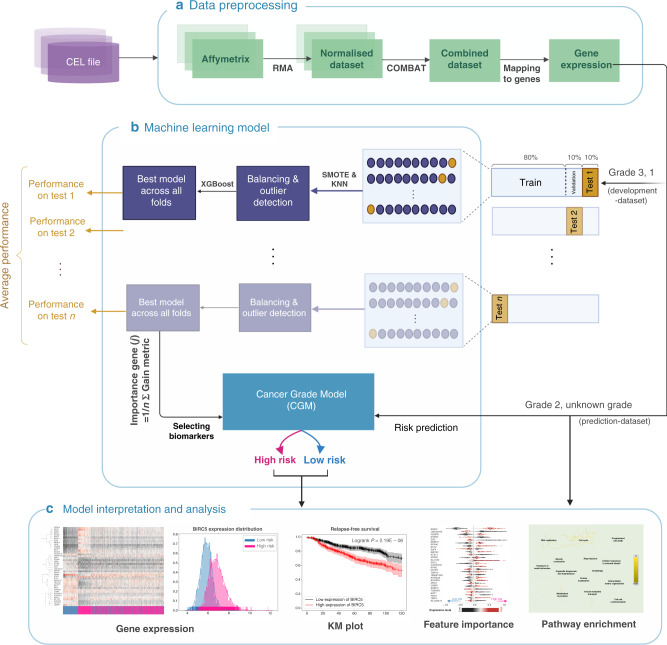
CGM overall computational framework. **a** Data pre-processing: input data comprise CEL files from different studies. For each dataset the Affymetrix probe intensity was normalised, datasets merged and batch effects removed. **b** Schematic representation of the CGM machine learning model. The XGBoost library was trained on grade-1 and grade-3 cancer samples over ten-fold cross-validation, which was repeated ten times. Feature importance based on average gain value was employed to reduce gene features to 70 markers with the best discriminatory properties. CGM was then applied to stratify grade-2 and unknown-grade samples into more or less aggressive cancer phenotypes (high-risk or low-risk cohorts, respectively). **c** Interpretation of machine learning model results. A series of analyses employing prognostic features, such as patient survival, gene importance based on SHAP values and pathway enrichment, were used to evaluate biomarkers and the stratification of cancer samples achieved by CGM.

Figure 1 shows the overall computational framework, which includes data pre-processing and integration, development of the CGM machine learning model and cross-validation, as well as key steps of model interpretation through feature prioritisation, prognostic data analysis and pathway enrichment.

### Dataset, pre-processing and integration

Gene expression data from 33 breast cancer datasets corresponding to platforms GPL570 [Genome U133 Plus 2.0] and GPL96 [Genome U133A] were obtained from Gene Expression Omnibus (Table 1) [22]. Samples with prior treatment were excluded (data selection workflow in Supplementary Figure S1). A total of 5031 tumour samples and 70 normal samples were collected along with their clinical characteristics, including ER, PR, and HER2 status, distant metastasis-free survival (DMFS), relapse-free survival (RFS), OS and PAM50 subtype [10] (Supplementary Table S1).

**Table 1:** List of GEO datasets employed in this study.

GEO ID	Platform	Sample type	# sample	# grade 1	# grade 2	# grade 3	# NA
GSE11121	GPL96	Primary tumour	200	29	136	35	0
GSE18864	GPL570	Tumour biopsy	84	10	16	58	0
GSE20711	GPL570	Primary tumour	88	13	5	70	0
GSE23593	GPL570	Primary tumour	50	2	23	25	0
GSE27120	GPL570	Primary tumour	28	3	11	14	0
GSE32646	GPL570	Tumour biopsy	115	16	78	21	0
GSE36771	GPL570	Unknown	107	11	42	54	0
GSE42568	GPL570	Primary tumour	104	11	40	53	0
GSE50948	GPL570	Primary tumour	154	0	68	86	0
GSE5460	GPL570	Primary tumour	129	27	32	70	0
GSE11001	GPL570	Primary tumour	30	4	13	13	0
GSE87007	GPL570	Primary tumour	31	5	3	23	0
GSE88770	GPL570	Primary tumour	117	13	96	7	1
GSE7390	GPL96	Primary tumour	198	30	83	83	2
GSE78958	GPL570	Primary tumour	424	88	156	178	2
GSE45255	GPL96	Primary tumour	139	17	52	67	3
GSE61304	GPL570	Tumour adjacent epithelium	62	5	16	37	4
GSE63471	GPL570	Tumour biopsy	142	5	52	81	4
GSE21653	GPL570	Primary tumour	266	45	89	125	7
GSE26639	GPL570	Tumour biopsy	226	15	83	121	7
GSE17907	GPL570	Primary tumour	55	3	10	34	8
GSE10810	GPL570	Primary tumour	32	2	10	10	10
GSE25066	GPL96	Primary tumour biopsy	508	32	180	259	37
GSE47109	GPL570	Primary tumour	246	43	115	49	39
GSE95700	GPL570	Primary tumour	57	0	0	0	57
GSE5327	GPL96	Primary tumour	58	0	0	0	58
GSE48390	GPL570	Primary tumour	81	0	0	0	81
GSE58984	GPL570	Unknown	94	0	0	0	94
GSE103091	GPL570	Primary tumour	104	0	0	0	104
GSE45827	GPL570	Primary tumour	130	0	0	0	130
GSE65194	GPL570	Primary tumour	130	0	0	0	130
GSE1456	GPL96	Primary Ttumour	159	0	0	0	159
GSE102484	GPL570	Primary tumour	683	0	0	0	683

Data integration comprised normalising raw intensity data in Affymetrix CEL data files through Robust Multichip Average [23] and removing batch effects with COMBAT [24] using R 3.3 and related libraries. Probes were mapped to genes, and in cases of multiple probes mapping to one gene, the average value was taken. The data processing pipeline is illustrated in Fig. 1a. For the implementation of the machine learning model and cross-validation, samples corresponding to grade 1 and grade 3 (henceforth termed *development-dataset*, 2002 samples) were used to build the classification model, which was later applied on grade-2 and unknown-grade samples (termed *prediction-dataset*, 3029 samples) to stratify them into low- and high-risk categories.

### Machine learning model development

The model was formulated as binary classification on the development-dataset, where gene expression values represented input variables and cancer grade 1 vs. grade 3 were output variables. An overview of the machine learning pipeline is shown in Fig. 1b. Model performance metrics were calculated through 10-fold cross-validation repeated ten times to prevent bias on the splitting dataset. The development-dataset was split into 80% training set, 10% validation set for hyperparameter tuning and developing the classifier, and 10% test set for evaluating performance metrics. Detecting outliers in train and validation datasets was performed with the *K*-nearest neighbour algorithm [25] of PyOD [26] library was used by measuring the distance of an observation to *k*th nearest neighbour as the outlying score. The relevant samples were removed from the training set. In cases of imbalanced data, over-sampling through Synthetic Minority Over-sampling Technique (SMOTE) [27] was used on the imbalanced dataset [13]. The machine learning model was built on XGBoost [28] (eXtreme Gradient Boosting), a machine learning method that combines weak learners (decision trees) to achieve stronger class discrimination.

Grid search was performed on training set samples within each cross-validation fold to find the best set of hyperparameters. The best-performing model was selected (termed CGM, with hyperparameters of maximum tree depth = 5, subsample ratio = 0.6, minimum child weight = 1, and gamma = 0.5 in XGBoost [28]) and applied to classify the prediction-dataset samples into high or low risk. For selecting genes that were most important in classification, the Gain metric [29] was used to calculate the average across all cross-validation sets. The smallest set of genes was identified, which reflected the highest Gain value and kept the model performance at the same level as and when all genes were employed. In addition, the SHAP [30] (SHapley Additive exPlanations) method was used to obtain features globally important for classification. Python 3.7.3, Scikit-learn 0.21.2 and XGBoost 0.90 were used to implement the models. Principal component analysis (PCA) [31] was applied to visualise the grouping of samples based on the expression of selected genes.

### Analysis and interpretation of machine learning prediction results

Clinical parameters (time and event of DMFS, RFS and OS) were used to evaluate differences between high- and low-risk groups in the development and prediction-dataset. Survival analysis was undertaken using Kaplan–Meier (KM) methodology [32]. For multivariable analysis, Cox’s proportional hazard model by CoxPHFitter [33] was used. Stratification was cross-referenced with PAM50 [10] and genomic tests for risk of metastasis and relapse (OncotypeDx [34], EndoPredict [35] and Gene expression Grade Index (GGI) developed in genefu [36] package in Bioconductor). Pathway enrichment was performed by mapping the selected biomarkers on Reactome [37] with *p* values calculated on a hypergeometric model [38] and a cut-off of 0.05. An overview is illustrated in Fig. 1c.

## Results

### Model training and risk prediction of grade-2 and unknown-grade samples

**Fig. 2:**
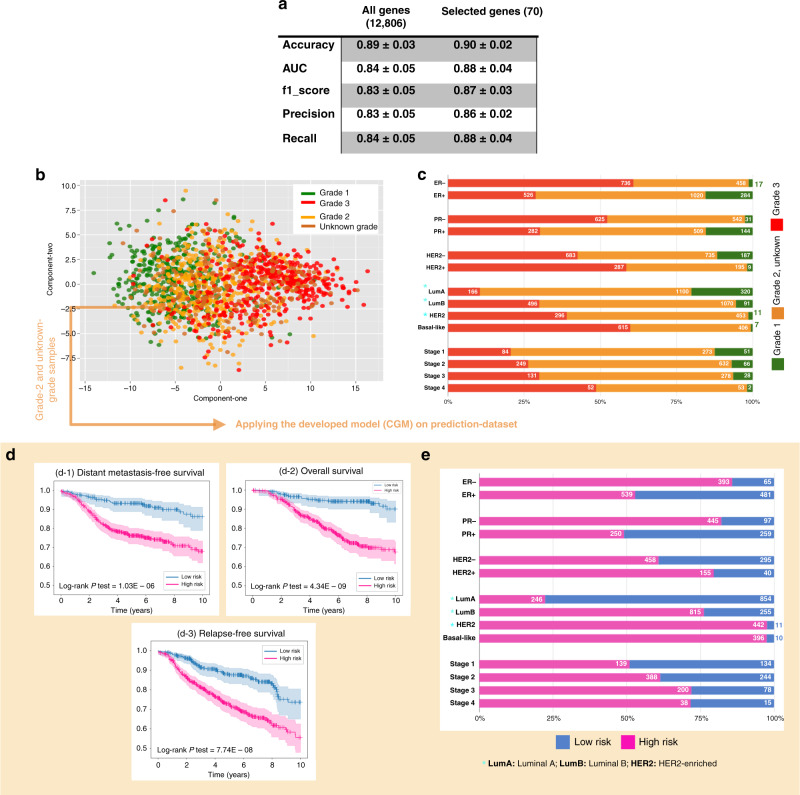
Model training and risk prediction. **a** Performance of the CGM methodology in classifying grade-1 and grade-3 samples. **b** Principal component analysis of samples across all three histological grades based on 70 biomarkers. **c** Hormone receptor characteristics and PAM50 subtype status of all samples. **d** Analysis through Kaplan–Meier plots across various survival metrics: distant metastasis-free survival (d-1), recurrence-free survival (d-2) and overall survival (d-3) for patients in the prediction-dataset (grade 2 and unknown grade) classified as either high risk (pink) or low risk (blue) by CGM. **e** Hormone receptor characteristics and PAM50 subtype status in high- and low-risk group samples.

Processed gene expression datasets used in this study were derived through the integration of 5031 samples (429 grade 1, 1409 grade 2, 1573 grade 3 and 1620 unknown grade) and 12,806 genes (Fig. 1a) spanning 33 breast cancer studies (Table 1). The dataset was divided into a *development-dataset* comprising the grade-1 and grade-3 samples (total of 2002) with which the machine learning model CGM was trained and tested, and a *prediction-dataset* of grade-2 and unknown-grade samples (total of 3029) for prediction and classification of these samples into low risk (1130) or high risk (1899). Clinical parameters and survival properties of the development and prediction datasets were used to validate the model. A machine learning model based on gradient boosted trees was built on grade-1 and grade-3 samples of the development-dataset (Fig. 1b), with a performance metric of 89% accuracy (Fig. 2a). Use of the Gain metric yielded a prioritisation list of gene features in terms of importance in the classification of grade-1 vs. grade-3 tumours. The 70 top genes with the highest Gain value (Supplementary Table S2) that kept model accuracy at the same level as all genes (Supplementary Figure S2) were selected and used for classification with model performance maintaining high accuracy of 90% (Fig. 2a).

For samples across all histological grades (including samples of unknown grade), PCA based on the expression of the 70 selected genes (Fig. 2b) illustrated that grade-3 samples separated well from milder phenotypes in grade 1, whereas grade-2 tumours reflected a widely diverging histological manifestation [3, 4]. Analysis of prognostic factors confirmed that grade-1 samples had substantially better survival outcome compared to grade 3, with grade 2 in mid-way (Supplementary Figure S3a). Clinical subtype information of the samples is shown in Fig. 2c. Most samples in high-grade disease (grade 3) were ER−, PR− and HER2+, while ER+, PR+ and HER2− groups were linked to low-grade samples (grade 1) mostly (Fig. 2c). There were also more grade-3 samples in the HER2-enriched and basal-like groups according to PAM50 molecular subtypes, compared with the less aggressive luminal A and luminal B samples. Importantly, in terms of both immunohistological status and PAM50 subtype, a large proportion of samples were grade 2 or unknown grade, which highlights the clinical challenge of assigning the right treatment for this group of patients and identifying means to dissect this diverse cancer cohort into more informative risk groups.

After building the XGBoost classifier on grade-1 and grade-3 samples, the CGM predictive framework was used to assign risk to samples in the prediction-dataset (grade-2/unknown-grade group, 3029 samples), thereby re-classifying these samples into high-risk (grade-3-like samples, 1899) or low-risk (grade-1-like samples, 1130). KM analysis for these groups (Fig. 2d) illustrated that RFS, DMFS and OS are significantly better in the low-risk group than in the high-risk group (log-rank *P* 1.03E − 06, 4.34E − 09 and 7.74E − 08, respectively). For instance, within 5 years, 5% of low-risk patients were reported to develop metastasis compared to >20% in the high-risk group (Fig. 2d–1). In terms of immunohistochemical subtypes (Fig. 2e), of samples classified as low risk in the prediction-dataset, 47% were ER+, 51% samples were PR+ and 40% HER2−, thereby representing cases where cytotoxic regimens can be avoided. According to the PAM50 subtype, of samples classified as high risk, 22% were luminal A, 76% luminal B, 98% HER2-enriched and 98% basal-like, linking them to cases benefiting from further systemic treatment.

Multivariable analysis was performed based on several clinical variables (hormone receptors, stage, age, etc.). The analysis showed the association between CGM, tumour stage and ER status with RFS (Supplementary Figure S4a). Therefore, we examined the joint distribution of stage, ER status and CGM. ER− status was generally associated with a high-risk group (grade-3 and CGM high-risk group, see Fig. 2c, e); however, ER-positive status was associated with a heterogeneous mixture of high- and low-risk groups, which CGM can stratify into better prognostic groups with log-rank *P* test = 1.95E − 10 (see Supplementary Figure S4c-1). Similarly, for stage 2 and stage 3, which represent intermediate risk, CGM can define prognostically relevant subgroups (log-rank *P* test = 1.36E − 03, and 1.36E − 03) (see Supplementary Figure S4c-2, 3).

### Evaluation of prognostically relevant biomarker genes

**Fig. 3:**
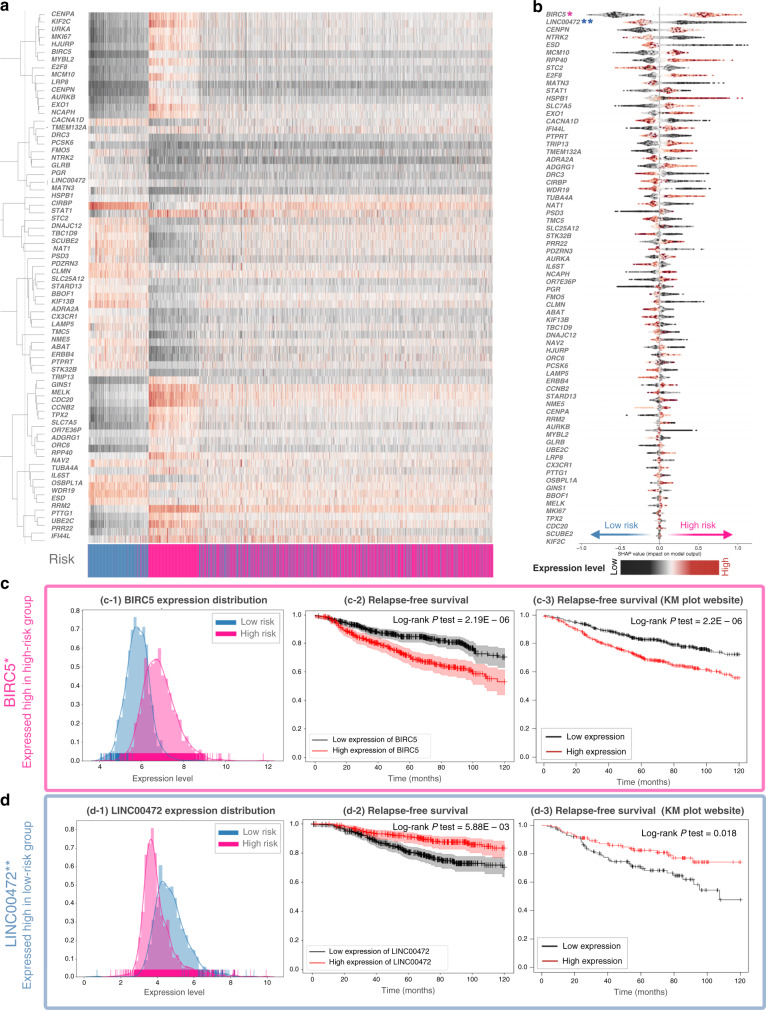
Analysis of the development dataset. **a** Hierarchical clustering of biomarker expression across the prediction-dataset (grade 2 and unknown grade). Samples are labelled according to risk assigned by CGM (pink for high risk and blue for low risk). **b** Prioritisation list according to SHAP values for the selected biomarker genes. Colour (black to red) indicates the values of expression of each gene feature, and the horizontal position of each point reflects the impact of that value in the prediction of risk (see text). **c**, **d** *BIRC5* and *LINC00472* as high- and low-risk genes, respectively (c-1, d-1). Expression value distribution for BIRC5 and *LINC00472* and in high- (pink) and low- (blue) risk groups. (c-2, d-2) *BIRC5* and *LINC00472* KM plots for relapse-free survival. (c-3, d-3) *BIRC5* and *LINC00472* KM plots for relapse-free survival are created by the KM plotter website based on grade-2 samples >120 months.

The machine learning model involved feature prioritisation through the use of the Gain value (Supplementary Table S2) and the 70 top-scoring genes were further evaluated through hierarchical clustering (Fig. 3a). Expression profile clusters showed association to sample risk labels, which are indicated by the grouping of samples (colour-coded columns according to predicted risk). A similar heatmap of gene expression that also included normal samples is shown in Supplementary Figure S5, which shows that low-risk tumours have not undergone significant gene expression changes when compared to normal breast tissues. Further prioritisation of genes was employed based on SHAP values [30] to determine the contribution of each gene feature in the predictions generated by the CGM framework (Fig. 3b). It is noted that (i) genes higher in the list reflect larger overall contribution to the prediction model, (ii) the colour of each data point indicates the expression value of the gene feature in the corresponding sample and (iii) the horizontal position of data points reflect the impact in prediction (i.e. high negative SHAP values show a stronger contribution to the prediction of low risk, whereas high positive values reflect the prediction of high risk). The same analysis employing hierarchical clustering and SHAP values for the development-dataset (grade-1 and grade-3 tumours) is shown in Supplementary Figure S3b, c.

Interpreting the prioritisation list in Fig. 3b can indicate cases of genes overexpressed in the high-risk group or overexpressed in low-risk samples that may be associated with molecules with important cancer-related activity. Mean expression values in the high- and low-risk groups for all 70 selected markers are shown in Supplementary Table S3. As an example, *BIRC5* (overexpressed in high-risk samples) and *LINC00472* (overexpressed in low-risk) are also shown to have significant prognostic value based on RFS calculated across our dataset and KM plotter (Fig. 3c, d). This observation is consistent with known roles for these genes, where high expression of *LINC00472* [39] is known to activate p53 signalling pathway that inhibits cancer development [40], while *BIRC5* encoding Survivin is involved in carcinogenesis by influencing cell division and proliferation and inhibiting apoptosis [41]. For other genes, *AURKA*, *PTTG1*, *CDC20*, *SLC7A5*, *E2F8*, *TPX2*, and *TUBA4A*, high expression in the high-risk group was linked to tumour growth and metastasis. On the other hand, increasing levels of *NME5* and *CACNA1D* expression could suppress malignant behaviour. Some of these biomarkers, including *E2F8* [42], *TPX2* [43] and *CACNA1D* [44], have been independently confirmed as tumourigenic or tumour-suppressive in breast cancer, and can thus point towards novel targets for treatment. Employing the Connectivity Map (CMap) [45] to search for potential drugs tested against the 70 marker genes indicated cases where some of these genes were explored as targets in clinical trials (details in Supplementary Table S4), demonstrating the potential of our findings.

Further validation of the 70 biomarkers indicated a significant association with survival outcome (survival calculation details in Supplementary Tables S5 and S6). Pathway enrichment analysis using the Reactome database indicated cell cycle regulation, gene expression and DNA replication as the most affected pathways for the high-risk group (Supplementary Figure S6a,c). Perturbation in any of these pathways was not observed in the low-risk group, indicating slow tumour growth and low metastatic potentials (Supplementary Figure S6b).

### Genomic tests for clinical assessment of breast cancer

**Fig. 4:**
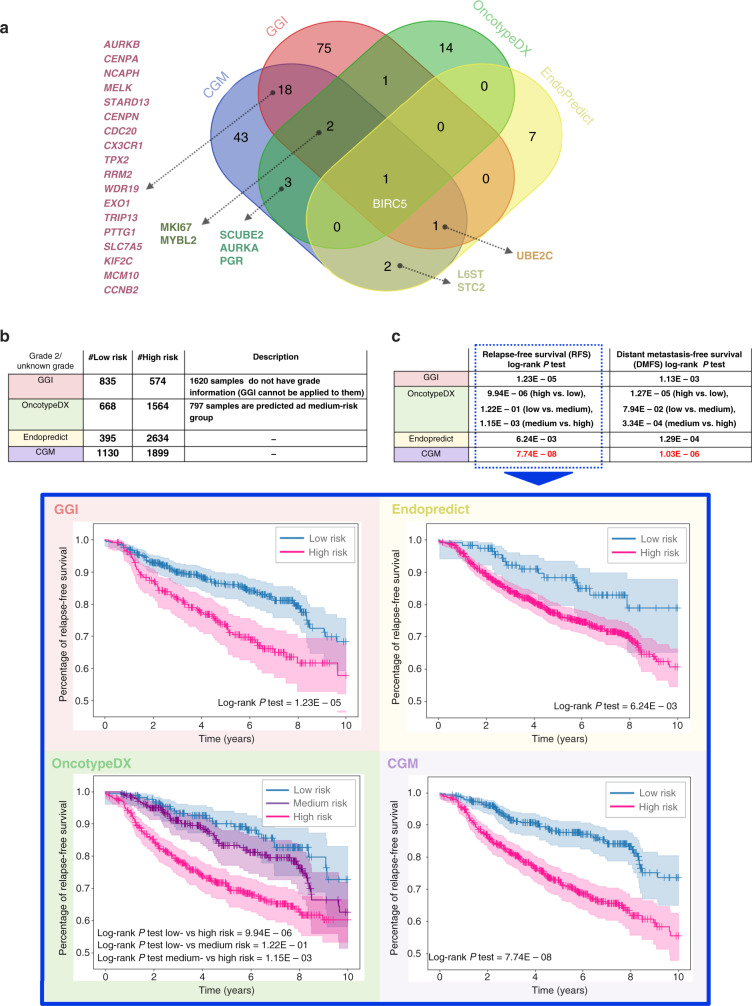
Comparison of CGM with existing genomic tests. **a** Venn diagram for comparison of gene biomarkers identified via CGM with other genomic test methods, namely GGI, OncotypeDX and Endopredict. **b** The number of samples in prediction-dataset (grade 2 and unknown grade) stratified to relevant risk groups through GGI, OncotypeDX, Endopredict and CGM. **c** Comparing GGI, OncotypeDX, Endopredict and CGM risk prediction on log-rank *P* test on distant metastasis- and relapse-free survival. Survival curves for relevant cohort predictions by each method are also shown.

Results of CGM on re-classifying grade-2 and unknown-grade tumours were compared to currently available genomic tests, namely OncotypeDX [34], EndoPredict [35], and GGI [4]. These tests associate gene activity level to cancer aggressiveness and are recommended in national and international guidelines for proposing adjuvant systemic therapy [6]. Venn diagram (Fig. 4a) showing overlapping biomarkers identified by CGM and the other reported genes by the three methods [4, 34, 35] (full gene list in Supplementary Table S7).

Pairwise comparison of CGM with each of the three methods (Fig. 4b and Supplementary Table S8) showed 91% similarity of sample classification with OncotypeDX (without considering samples predicted in medium-risk group by OncotypeDX), followed by EndoPredict and GGI with 76% and 74% similarity, respectively. It is noted that as GGI does not work on unknown-grade samples, those samples were eliminated for comparison with GGI. We assessed survival through KM plots for DMFS and RFS on the prediction-dataset with all four methods and showed that CGM was more discriminative in prognosis and better in classifying samples into the high- or low-risk groups, as indicated by the relevant *P* values (Fig. 4c and Supplementary Figure S7). We also performed the multivariable analysis of the prognostic methods (GGI, OncotypeDX and EndoPredict) based on time to relapse using fitting Cox’s proportional hazard model in Supplementary Figure S4b, which shows the strongest association of CGM with RFS (hazard ratio (HR) = 1.71, 95% confidence interval (CI) = 1.2–2.43; *P* < 0.005).

## Discussion

Breast cancer is a heterogeneous disease with significant variance in genetic predisposition and phenotypic characteristics. Despite progress, assigning a more accurate prognosis requires optimisation and significant challenges remain in attaching appropriate treatment to relevant patient subgroups. It is widely accepted that tumour classification based on intrinsic features such as histological grade can predict prognostic features or treatment options more accurately than time-dependent factors [1, 3]. A case in point is the prescription of chemotherapy in grade-3 tumours, but not in those of grade 1. However, for grade-2 phenotypes that represent a heterogeneous cancer class, prognosis and treatment remain particularly challenging with either over-treatment or missed diagnoses being common. To help address these limitations, here we report CGM a machine learning platform based on gradient boosted trees to classify grade-2 and unknown-grade tumours into high and low risk, after training the model on genomic data of high- and low-grade cancers and generating a 70-gene signature. Our methodology includes the use of feature importance metrics such as Gain and SHAP values, which offer means of attributing biological significance to specific genes and are particularly important in counter-balancing the black-box nature of machine learning models.

Based on the 70 genes selected as markers, the CGM prediction model can successfully separate grade-2 and unknown-grade samples into high- or low-risk groups, regardless of the conventional surface receptor immunohistochemistry-based subtypes or the PAM50 molecular-based subgrouping. Basal-like and HER2-enriched subtypes are high-risk tumours, and, in the absence of treatment, these patients have a poor prognosis [46]. In the clinic, the treatment strategy for HER2-enriched breast cancers is mainly by targeted therapy with trastuzumab in combination with chemotherapy, while basal-like/TNBC are treated with radiotherapy and chemotherapy until the recent development of olaparib for BRCA-mutated TNBC, and anti-PD-L1 immunotherapy in combination with chemotherapy for advanced disease [47, 48]. In our predictive model, 98% of HER2-enriched and 98% of basal-like grade 2/unknown-grade tumours are classified as high-risk and would require chemotherapy, in line with current disease control settings for these two tumour types. However, prediction via our CGM model would benefit patients in groups classified as less aggressive.

We evaluated the prognostic power of the CGM model and found that among patients with grade-2/unknown-grade cancer, about half of those with ER/PR tumour expression and 22% of patients with luminal A breast cancer have high-risk disease. The CGM model can determine the potential prognosis of patients based on their genetic signature and can indicate whether a patient with grade 2 or unknown grade has a high-risk disease and thus may require immediate treatment intervention or whether they have a low-risk disease and may benefit from less aggressive treatment strategies [49]. In clinical practice, our model is unlikely to be applied to very aggressive subtypes such as HER2-overexpressed and TNBC, but to subtypes that have a more “intermediate” prognosis such as luminal B. Treatment of luminal B cancers is typically based on an in-depth list of criteria, including size, lymph node involvement, grade, Ki67 status and a low personal risk of relapse determined by other gene expression signature models such as OncotypeDX [50]. It is possible that our CGM model may be used to determine the potential prognosis of these patients, and thus inform treatment strategy independent of clinical characteristics.

Our model also revealed genes that may contribute towards cancer progression. Some of these are known to be associated with breast cancer, while others are less well understood and would benefit from further functional characterisation. Literature search and pathway analysis using Reactome [51] identified key functions and pathways associated with our biomarkers. These mostly associated with the cell cycle, DNA replication, transcription, and signal transduction. Furthermore, while the high-risk gene set involved multiple genes connecting the above pathways, the low-risk gene set had no pathways with more than one gene affected (Supplementary Figure S6). This indicates that those samples feature low cancer proliferation rates, lack of escape mechanisms and lower metastatic potential, and could hence be classified as low-risk cancers that might not require systemic and cytotoxic therapies.

Although unsurprisingly the most fundamental traits identified for a high-risk group were predominantly related to the dysregulation of cell cycle checkpoints and transcription regulator molecules [52, 53], individual genes could also provide insights for novel treatment development. Downregulation of *BIRC5* (survivin), which is the top-ranked biomarker revealed by CGM, was reported as an inhibitor of tumour cell migration and invasion through the PI3K/Akt signalling pathway [54]. Survivin remains a promising target for drug discovery and breast cancer therapeutics, ranging from selective suppressants that disrupt survivin function [55] to antisense oligonucleotides that degrade survivin messenger RNA [56]. Survivin peptide-mediated immunotherapy has also been tested in clinical trials [57]. An interaction between *AURKA* (Aurora Kinase A) and MAPK pathway has been proposed for a new treatment strategy using a combination of AURKA and MEK1/2 inhibitors in breast cancer [58, 59]. PTTG1 contributes to different cancer-promoting pathways that can increase cell growth through a nuclear exclusion of p27 [60]. CDC20 is overexpressed in TNBC and could be used as a treatment target [61], while the expression of SLC7A5 part of the large neutral amino acid transporter small subunit 1 heterodimer has been reported to correlate with luminal cancers [62], and anti-SLC7A5 targeted therapies have been developed for endocrine therapy-resistant tumours [63]. On the other hand, low expression of LINC00472, thought to act as a tumour suppressor, has been reported to suppress nuclear factor-κB signalling [39], which contributes to tumour progression and metastasis [64]. NME5 plays a key role in DNA proofreading and repair [65] and would be predicted to be associated with low-risk cancers.

We further compared CGM with well-known diagnostic methods in the literature [6] to validate the predictive power of our model. OncotypeDX and EndoPredict predict distant recurrence of early-stage breast cancer (stage 1 and 2) based on a set of 21 and 11 genes, respectively (Supplementary Table S7). OncotypeDX [34] calculates a recurrence score between 0 and 100 to reflect the likelihood of breast cancer recurrence within 10 years and to classify patients into groups of low (<26), medium (26–30) and high (>30) score, where higher scores indicate a greater likelihood of recurrence. EndoPredict [35] analyses tumour gene activation to provide a risk score for ER/PR+, HER2− samples as either low risk or high risk, and recurring as distant metastasis within 10 years. Finally, GGI [4] employs 97 differentially expressed genes between histologic grade-1 and grade-3 tumours, which are selected through the analysis of 189 breast cancer microarray datasets to classify grade-2 tumours into two groups (grade 1 or grade 3) to suggest relevant treatment.

Unlike CGM, OncotypeDX classifies patients into three groups (low risk, medium risk or high risk), which leads to the assignment of samples into a non-informative medium-risk group category, while the difference between low- and medium-risk groups is not significant (Fig. 4c). Unlike CGM, GGI requires the grade of the tumours to be known a priori, which may not always be available and relies on parameters calculated for the given dataset (its scale and offset), thus making it dependent on the dataset used. Furthermore, GGI ignores the importance of genes in cancer by assigning equal weight to all of them and relying on their sum. This can lead to a lack of interpretability and may prevent understanding of the individual contribution of each gene in aggressive tumours. EndoPredict does not employ a medium group; however, it tends to place more patients into the high-risk group (about 7 times more than low risk, Fig. 4b). There are about twice more patients in the low-risk groups in the two other methods (668 by OncotypeDX and 835 by GGI) compared to EndoPredict (395), suggesting that EndoPredict tends to a larger number of high-risk predictions (395 low risk and 2634 high risk), thereby leading to over-treatment.

In overview, moderately differentiated tumours represented by those diagnosed as grade 2 are particularly difficult to treat, leading to over- or under-treatment in this patient group. Genomic information can identify novel predictive biomarkers and signalling pathways indicative of disease progression or phenotype. In this study, we present a computational pipeline using gradient boosted trees to analyse large and complex datasets, integrated from multiple breast cancer studies, to discover patient subtypes and derive an understanding of prognosis. Even though we exemplify our platform on gene expression data for histological grade prediction, our strategy is generic and can be applied to other types of high-throughput data and clinical labels.

Our model can assign high- and low-risk groups, without using clinical data such as tumour size, stage or breast cancer subgroup information, offering a potential means to avoiding underdiagnosis of high-risk tumours and minimise over-treatment of low-risk diseases, thus helping to reduce unnecessary toxic treatments. In addition, our findings of key genes differentiating patient phenotypes may point to important regulators of aggressive disease phenotype and lead to a better understanding of underlying malignant disease mechanisms across subgroups. These could ultimately point to novel therapeutic targets applicable to specific disease types. Even though CGM is implemented and evaluated on breast cancer, capitalising on the rich and well-studied datasets for this cancer, we stress that our methodology can be used in other types of cancer where subtypes are less well understood, and the application of well-established machine learning methods would add valuable prognostic insights.

## Supplementary information

Supplementary Table S1

Supplementary Table S2

Supplementary Table S3

Supplementary Table S4

Supplementary Table S5

Supplementary Table S6

Supplementary Table S7

Supplementary Table S8

Supplementary Figure Legends

Supplementary Figure S1

Supplementary Figure S2

Supplementary Figure S3

Supplementary Figure S4

Supplementary Figure S5

Supplementary Figure S6

Supplementary Figure S7

## Data Availability

The datasets that support the findings of this study are collected from the Gene Expression Omnibus (GEO) repository [http://www.ncbi.nlm.nih.gov/geo].

## Code availability

The CGM code will be made available at: https://github.com/elmira-amiri/CGM.
