# Supplementary material for: Cancer Grade Model: a multi-gene machine learning-based risk classification for improving prognosis in breast cancer
Source: Br J Cancer. 2021 Jun 15;125(5):748–58. doi: 10.1038/s41416-021-01455-1 (PMC8405688; doi:10.1038/s41416-021-01455-1)
Supplement: Supplementary file 1 — Supplementary Table S1 [file 41416_2021_1455_MOESM1_ESM.pdf]

Table S1: Clinical characteristics and number of tumour samples

Grade	#	PAM50	#
grade-1	429	luminal A	1586
grade-2	1409	luminal B	1657
grdae-3	1573	basal-like	1075
NA	1620	HER2-positive	713
**ER**	**#**	**PR**	**#**
positive	1830	positive	935
negative	1211	negative	1198
NA	1990	NA	2898
**HER2**	**#**	**DISTANT METASTASIS (event)**	**#**
positive	491	No	1427
negative	1623	Yes	356
NA	2917	NA	3248
**RELAPSE (event)**	**#**	**OVERALL SURVIVAL (event)**	**#**
No	1149	Alive	762
Relapse	465	Death	222
NA	3417	NA	4047
**Stage**	**#**	**Age**	
stage-1	408	>= 50	1090
stage-2	947	< 50	1084
stage-3	437	NA	2857
stage-4	107		
NA	3132		
