# Supplementary material for: Cancer Grade Model: a multi-gene machine learning-based risk classification for improving prognosis in breast cancer
Source: Br J Cancer. 2021 Jun 15;125(5):748–58. doi: 10.1038/s41416-021-01455-1 (PMC8405688; doi:10.1038/s41416-021-01455-1)
Supplement: Supplementary file 2 — Supplementary Table S2 [file 41416_2021_1455_MOESM2_ESM.pdf]

Table S2: The 70 top genes with the highest Gain value

CGM genes	EntrezGeneID	Gain-value	CGM genes	EntrezGeneID	Gain-value
BIRC5	332	28848.55	LRP8	7804	1322.02
CDC20	991	14277.15	IL6ST	3572	1311.01
CCNB2	9133	13534.73	PGR	5241	1295.24
PTTG1	9232	13528.45	OSBPL1A	114876	1206.25
AURKA	6790	13237.34	TMC5	79838	1204.91
SLC7A5	8140	9167.33	ORC6	23594	1200.26
TPX2	22974	8364.85	CIRBP	1153	1179.25
CENPN	55839	8026.90	STAT1	6772	1140.72
MCM10	55388	6084.97	ESD	2098	1137.05
UBE2C	11065	4787.60	MYBL2	4605	1090.52
NME5	8382	4115.41	PRR22	163154	1084.95
MELK	9833	4078.71	CLMN	79789	1047.54
CENPA	1058	3946.02	NCAPH	29781	1044.98
KIF2C	11004	3718.10	PDZRN3	23024	1013.13
TRIP13	9319	2925.61	TUBA4A	7277	978.82
SCUBE2	57758	2828.51	DNAJC12	56521	953.06
STC2	8614	2748.84	CX3CR1	1524	948.77
PTPRT	11122	2464.10	IFI44L	10964	936.29
LINC00472	79940	2447.34	TBC1D9	23061	935.21
STK32B	55351	2431.15	HSPB1	3315	892.66
RRM2	6241	2335.24	TMEM132A	54972	883.82
BBOF1	80127	2334.78	PSD3	23362	875.81
EXO1	9156	2277.14	NTRK2	4915	857.44
MKI67	4288	2257.93	OR7E36P	26637	853.79
WDR19	57728	2179.16	GLRB	2743	845.05
E2F8	79733	2168.62	STARD13	90627	842.20
AURKB	9212	2060.70	ABAT	18	832.26
DRC3	83450	1971.17	MATN3	4148	811.27
NAT1	9	1949.96	ADRA2A	150	792.54
ERBB4	2066	1943.43	ADGRG1	9289	763.06
HJURP	55355	1764.60	NAV2	89797	739.07
KIF13B	23303	1491.30	FMO5	2330	734.62
PCSK6	5046	1491.12	GINS1	9837	734.48
CACNA1D	776	1463.04	RPP40	10799	723.39
SLC25A12	8604	1457.68	LAMP5	3911	714.39
