# Supplementary material for: Cancer Grade Model: a multi-gene machine learning-based risk classification for improving prognosis in breast cancer
Source: Br J Cancer. 2021 Jun 15;125(5):748–58. doi: 10.1038/s41416-021-01455-1 (PMC8405688; doi:10.1038/s41416-021-01455-1)
Supplement: Supplementary file 3 — Supplementary Table S3 [file 41416_2021_1455_MOESM3_ESM.pdf]

Table S3: Mean expression values in the high and low risk groups for selected genes

Gene*	Mean expression in low-risk**	Mean expression in high-risk**
BIRC5	5.84	6.88
LINC00472	4.78	4.00
CENPA	5.73	6.65
NTRK2	5.62	5.04
ESD	8.05	7.80
MCM10	4.61	5.84
RPP40	6.94	7.33
STC2	8.95	7.44
E2F8	4.35	5.39
MATN3	4.87	4.07
STAT1	8.88	9.68
HSPB1	10.69	10.81
SLC7A5	6.77	8.03
EXO1	5.20	6.11
CACNA1D	6.38	5.61
IFI44L	7.61	8.48
PTPRT	6.59	5.69
TRIP13	6.30	7.39
TMEM132A	5.71	5.97
ADRA2A	7.16	6.24
ADGRG1	7.30	7.69
DRC3	5.92	5.36
CIRBP	10.31	9.64
WDR19	7.90	7.28
TUBA4A	7.98	8.37
NAT1	10.10	8.38
PSD3	7.32	6.38
TMC5	7.30	6.03
SLC25A12	7.18	6.74
STK32B	6.88	6.12
PRR22	7.11	7.83
PDZRN3	7.79	7.00
AURKA	5.61	6.48
IL6ST	8.60	7.72
NCAPH	5.27	6.09

Gene	Mean expression in low-risk	Mean expression in high-risk
OR7E36P	7.27	7.81
PGR	5.51	4.41
FMO5	5.92	5.04
CLMN	7.22	6.81
ABAT	7.26	6.32
KIF13B	7.94	7.15
TBC1D9	9.43	8.51
DNAJC12	8.95	7.52
NAV2	7.82	7.35
HJURP	5.60	6.49
ORC6	6.70	7.40
PCSK6	5.67	5.38
LAMP5	7.44	6.74
ERBB4	6.55	5.56
CCNB2	6.56	7.79
STARD13	7.56	6.88
NME5	6.85	5.79
CENPN	5.08	5.80
RRM2	7.25	8.70
AURKB	5.13	6.04
MYBL2	6.23	6.98
GLRB	5.54	4.78
UBE2C	7.45	8.76
LRP8	5.08	5.89
CX3CR1	7.86	6.57
PTTG1	7.92	9.03
OSBPL1A	8.54	8.04
GINS1	6.52	7.58
BBOF1	7.67	6.80
MELK	6.74	7.99
MKI67	5.70	6.57
TPX2	6.32	7.64
CDC20	6.33	7.69
SCUBE2	9.89	7.79
KIF2C	5.77	6.73

*__pink:__ expressed high (colored red) in the high-risk group, **blue:** expressed low (colored black) in the high-risk group based on mean expression level in high- and low-risk group

** Normalized expression level
