# Supplementary material for: Cancer Grade Model: a multi-gene machine learning-based risk classification for improving prognosis in breast cancer
Source: Br J Cancer. 2021 Jun 15;125(5):748–58. doi: 10.1038/s41416-021-01455-1 (PMC8405688; doi:10.1038/s41416-021-01455-1)
Supplement: Supplementary file 4 — Supplementary Table S4 [file 41416_2021_1455_MOESM4_ESM.pdf]

Table S4: Potential drugs tested against the selected genes

Name	MOA	Target	Disease Area	Phase	Name	MOA	Target	Disease Area	Phase
YM-155	survivin inhibitor	BIRC5		Phase 2	fluticasone-propionate	glucocorticoid receptor agonist	PGR	dermatology	Launched
terameprocol	lipoxygenase inhibitor	BIRC5		Phase 1/Phase 2	gestrinone	progesterone receptor antagonist	PGR	obstetrics/gynecology	Launched
amitriptyline	norepinephrine inhibitor, norepinephrine reuptake inhibitor	NTRK2, ADRA2A	neurology/psychiatry	Launched	hydroxyprogesterone	progesterone receptor agonist	PGR	obstetrics/gynecology	Launched
ANA-12	tropomyosin receptor kinase inhibitor	NTRK2		Preclinical	hydroxyprogesterone-acetate	progesterone receptor agonist	PGR		Launched
entrectinib	ALK tyrosine kinase receptor inhibitor, proto-oncogene tyrosine protein kinase inhibitor	NTRK2	oncology	Launched	medroxyprogesterone-acetate	progesterone receptor agonist	PGR	endocrinology	Launched
GNF-5837	growth factor receptor inhibitor	NTRK2		Preclinical	norelgestromin		PGR	endocrinology	Launched
larotrectinib	tropomyosin receptor kinase inhibitor	NTRK2		Launched	norethindrone	progesterone receptor agonist	PGR	obstetrics/gynecology	Launched
LM-22A4	tropomyosin receptor kinase inhibitor	NTRK2		Preclinical	norethindrone-acetate	progesterone receptor agonist	PGR	endocrinology, orthopedics, obstetrics/gynecology	Launched
glutathione	antioxidant	ESD		Phase 3	noretynodrel	progestogen hormone	PGR	endocrinology	Launched
CKD-712	NFkB pathway inhibitor	STAT1		Phase 1	17-hydroxyprogesterone-caproate	progesterone receptor agonist	PGR	obstetrics/gynecology	Launched
SB-242235	p38 MAPK inhibitor	HSPB1		Phase 1	megestrol-acetate	progesterone receptor agonist	PGR	neurology/psychiatry, endocrinology	Launched
bicyclol	NFkB pathway inhibitor	HSPB1	infectious disease	Launched	chlormadinone-acetate	5 alpha reductase inhibitor	PGR	endocrinology	Launched
cleviprex	calcium channel blocker	CACNA1D	cardiology	Launched	valproic-acid	benzodiazepine receptor agonist, HDAC inhibitor	ABAT	neurology/psychiatry	Launched
dronedarone	adrenergic receptor antagonist	CACNA1D, ADRA2A	cardiology	Launched	vigabatrin	GABA aminotransferase inhibitor	ABAT	neurology/psychiatry	Launched
gabapentin	calcium channel blocker	CACNA1D	infectious disease, neurology/psychiatry	Launched	2-oxopropanoate	pyruvate dehydrogenase kinase inhibitor	ABAT		Preclinical
isradipine	calcium channel blocker	CACNA1D	cardiology	Launched	3-methyl-GABA	GABA aminotransferase activator	ABAT		Preclinical
manidipine	calcium channel blocker	CACNA1D	cardiology	Launched	C11-Acetate		ABAT		Phase 3
mibefradil	T-type calcium channel blocker	CACNA1D		Withdrawn	L-alanine		ABAT		Launched
nifedipine	calcium channel blocker	CACNA1D, GLRB	cardiology	Launched	L-glutamic-acid	glutamate receptor agonist	ABAT		Launched
nilvadipine	calcium channel blocker	CACNA1D	cardiology	Launched	MC-1		ABAT		Phase 3
nisoldipine	calcium channel blocker	CACNA1D	cardiology	Launched	phenelzine	monoamine oxidase inhibitor	ABAT	neurology/psychiatry	Launched
nitrendipine	calcium channel blocker	CACNA1D	cardiology	Launched	poziotinib	EGFR inhibitor	ERBB4		Phase 2
sipatrigine	voltage-gated sodium channel blocker	CACNA1D		Phase 2	WAY-200070	estrogen receptor agonist	ERBB4		Preclinical
SNAP-5089	adrenergic receptor antagonist	CACNA1D,ADRA2A		Preclinical	AEE788	EGFR inhibitor, VEGFR inhibitor	ERBB4		Phase 1/Phase 2
amlodipine	calcium channel blocker	CACNA1D	cardiology	Launched	BMS-599626	EGFR inhibitor, protein tyrosine kinase inhibitor	ERBB4		Phase 1
spironolactone	mineralocorticoid receptor antagonist	CACNA1D, PGR	endocrinology, cardiology, rheumatology	Launched	canertinib	EGFR inhibitor	ERBB4		Phase 3
verapamil	calcium channel blocker	CACNA1D	cardiology	Launched	dacomitinib	EGFR inhibitor	ERBB4		Launched
WB-4101	adrenergic receptor antagonist	ADRA2A		Preclinical	vandetanib	EGFR inhibitor, RET tyrosine kinase inhibitor, VEGFR inhibitor	ERBB4	oncology	Launched
xylazine	adrenergic receptor agonist	ADRA2A	neurology/psychiatry	Launched	afatinib	EGFR inhibitor	ERBB4	oncology	Launched
yohimbine	adrenergic receptor antagonist	ADRA2A	cardiology	Launched	cladribine	adenosine deaminase inhibitor, ribonucleotide reductase inhibitor	RRM2	hematologic malignancy	Launched
ziprasidone	dopamine receptor antagonist, serotonin receptor antagonist	ADRA2A	neurology/psychiatry	Launched	clofarabine	ribonucleotide reductase inhibitor	RRM2	hematologic malignancy	Launched
zuclopenthixol	dopamine receptor antagonist	ADRA2A	neurology/psychiatry	Launched	fludarabine	ribonucleotide reductase inhibitor	RRM2	hematologic malignancy	Launched
2-BFI	imidazoline receptor ligand	ADRA2A		Preclinical	gemcitabine	ribonucleotide reductase inhibitor	RRM2	oncology	Launched
cabazitaxel	microtubule inhibitor	TUBA4A	oncology	Launched	hydroxyurea	ribonucleotide reductase inhibitor	RRM2	hematologic malignancy, oncology	Launched
colchicine	microtubule inhibitor	TUBA4A	rheumatology, endocrinology	Launched	triapine	ribonucleotide reductase inhibitor	RRM2		Phase 2
docetaxel	tubulin polymerization inhibitor	TUBA4A	oncology	Launched	fludarabine-phosphate	ribonucleotide reductase inhibitor	RRM2	hematologic malignancy	Launched
paclitaxel	tubulin polymerization inhibitor	TUBA4A	oncology	Launched	hesperadin	Aurora kinase inhibitor	AURKB		Preclinical
podophyllotoxin	microtubule inhibitor, tubulin polymerization inhibitor	TUBA4A	infectious disease	Launched	hesperidin	flavanone glycoside	AURKB		Launched
vincristine	tubulin polymerization inhibitor	TUBA4A	hematologic malignancy	Launched	reversine	Aurora kinase inhibitor	AURKB		Preclinical
vinorelbine	tubulin polymerization inhibitor	TUBA4A	oncology	Launched	TAK-901	Aurora kinase inhibitor	AURKB		Phase 1
L-aspartic-acid	metallic radical formation stimulant	SLC25A12		Launched	barasertib-HQPA	Aurora kinase inhibitor	AURKB		Phase 2/Phase 3
MK-5108	Aurora kinase inhibitor	AURKA, AURKB		Phase 1	CGP-57380	MAP kinase inhibitor	AURKB		Preclinical
MK-8745	Aurora kinase inhibitor	AURKA		Preclinical	enflurane	membrane permeability inhibitor	GLRB	neurology/psychiatry	Launched
MLN8054	Aurora kinase inhibitor	AURKA		Phase 1	gavestinel	glutamate receptor antagonist	GLRB		Phase 3
orantinib	FGFR inhibitor, PDGFR tyrosine kinase receptor inhibitor, VEGFR inhibitor	AURKA, AURKB		Phase 3	ginkgolide-A	GABA receptor antagonist	GLRB		Preclinical
PF-03814735	Aurora kinase inhibitor	AURKA, AURKB		Phase 1	halothane	glutamate receptor antagonist	GLRB	neurology/psychiatry	Launched
PHA-680632	Aurora kinase inhibitor	AURKA, AURKB		Preclinical	isoflurane	inhaled anaesthetic	GLRB	neurology/psychiatry	Launched
SNS-314	Aurora kinase inhibitor	AURKA, AURKB		Phase 1	lindane		GLRB	infectious disease	Launched
TC-A-2317	Aurora kinase inhibitor	AURKA		Preclinical	methoxyflurane	membrane permeability inhibitor	GLRB	neurology/psychiatry	Launched
tozasertib	Aurora kinase inhibitor, Bcr-Abl kinase inhibitor, FLT3 inhibitor, JAK inhibitor	AURKA, AURKB		Phase 2	ORG-25543	glycine transporter inhibitor	GLRB		Preclinical
ZM-447439	Aurora kinase inhibitor	AURKA, AURKB		Preclinical	picrotin	GABA receptor antagonist	GLRB		Phase 2
alisertib	Aurora kinase inhibitor	AURKA		Phase 3	picrotoxinin	GABA receptor antagonist	GLRB		Preclinical
AMG900	Aurora kinase inhibitor	AURKA, AURKB		Phase 1	sevoflurane	membrane integrity inhibitor	GLRB	neurology/psychiatry	Launched
AT-9283	Aurora kinase inhibitor, JAK inhibitor	AURKA, AURKB		Phase 2	strychnine	acetylcholine receptor antagonist	GLRB		Preclinical
aurora-a-inhibitor-i	Aurora kinase inhibitor	AURKA		Preclinical	thiocolchicoside	GABA receptor antagonist	GLRB	neurology/psychiatry	Launched
barasertib	Aurora kinase inhibitor	AURKA, AURKB		Phase 2/Phase 3	tropisetron	serotonin receptor antagonist	GLRB	gastroenterology	Launched
BI-847325	Aurora kinase inhibitor, MEK inhibitor	AURKA, AURKB		Phase 1	bilobalide	GABA receptor modulator	GLRB		Preclinical
CCT129202	Aurora kinase inhibitor	AURKA, AURKB		Preclinical	ginkgolide-B	platelet activating factor receptor antagonist	GLRB		Phase 3
CCT137690	Aurora kinase inhibitor	AURKA, AURKB		Preclinical	OTS167	maternal embryonic leucine zipper kinase inhibitor	MELK		Phase 1/Phase 2
CYC116	Aurora kinase inhibitor	AURKA, AURKB		Phase 1	nomegestrol-acetate	progesterone receptor agonist	PGR	endocrinology	Launched
danusertib	Aurora kinase inhibitor, growth factor receptor inhibitor	AURKA, AURKB		Phase 2	norgestimate	progesterone receptor agonist	PGR	endocrinology	Launched
ENMD-2076	Aurora kinase inhibitor, FLT3 inhibitor, VEGFR inhibitor	AURKA		Phase 2	norgestrel	progesterone receptor agonist	PGR	endocrinology	Launched
GSK1070916	Aurora kinase inhibitor	AURKA, AURKB		Phase 1	progesterone	progesterone receptor agonist	PGR	obstetrics/gynecology, endocrinology	Launched
GSK2334470	phosphoinositide dependent kinase inhibitor	AURKA, AURKB		Preclinical	segesterone-acetate	progesterone receptor agonist	PGR		Launched
JNJ-7706621	CDK inhibitor	AURKA, AURKB		Preclinical	tanaproget	progesterone receptor agonist	PGR		Phase 2
KW-2449	Abl kinase inhibitor, Aurora kinase inhibitor, FLT3 inhibitor	AURKA, AURKB		Phase 1	telaprevir	HCV inhibitor	PGR	infectious disease	Launched
levonorgestrel	estrogen receptor agonist, progesterone receptor agonist	PGR	endocrinology	Launched	ulipristal	progesterone receptor antagonist	PGR	endocrinology	Launched
medroxyprogesterone	progesterone receptor agonist	PGR	endocrinology	Launched	allylestrenol	steroidal progestin	PGR	obstetrics/gynecology	Launched
mifepristone	glucocorticoid receptor antagonist, progesterone receptor antagonist	PGR	endocrinology	Launched	altrenogest	progestogen hormone	PGR	endocrinology	Launched
dienogest	progesterone receptor agonist	PGR	endocrinology, obstetrics/gynecology	Launched	cridanimod	progesterone receptor agonist	PGR	infectious disease	Launched
drospirenone	mineralocorticoid receptor antagonist	PGR	endocrinology	Launched	danazol	estrogen receptor antagonist, progesterone receptor agonist	PGR	obstetrics/gynecology, cardiology	Launched
dydrogesterone	progesterone receptor agonist	PGR	obstetrics/gynecology	Launched	desogestrel	progesterone receptor agonist	PGR	endocrinology	Launched
ethynodiol-diacetate		PGR	endocrinology	Launched	etonogestrel	progesterone receptor agonist	PGR	endocrinology	Launched
