# Supplementary material for: Cancer Grade Model: a multi-gene machine learning-based risk classification for improving prognosis in breast cancer
Source: Br J Cancer. 2021 Jun 15;125(5):748–58. doi: 10.1038/s41416-021-01455-1 (PMC8405688; doi:10.1038/s41416-021-01455-1)
Supplement: Supplementary file 5 — Supplementary Table S5 [file 41416_2021_1455_MOESM5_ESM.pdf]

Table S5: Survival analysis for genes identified as over-expressed in high-risk group (with high Hazard Ratio values)

Genes affecting Metastasis, Relapse, and Overall survival	10yr OS		10yr RFS		10yr DMFS	
**geneID**	HR_exp(coef)	*p* longrank	HR_exp(coef)	*p* longrank	HR_exp(coef)	*p* longrank
ADGRG1	2.823	****	2.105	****	1.619	**
KIF2C	2.690	****	2.033	****	1.478	*
UBE2C	2.586	****	1.600	**	1.880	***
CDC20	2.579	****	1.974	****	1.616	**
CCNB2	2.497	****	1.949	****	1.872	***
AURKB	2.493	****	1.653	***	1.890	***
PTTG1	2.327	****	1.859	****	1.403	*
BIRC5	2.265	****	1.954	****	1.662	**
TPX2	2.213	****	1.786	****	1.522	*
TRIP13	2.185	****	1.859	****	1.515	*
AURKA	2.179	****	1.819	****	1.480	*
TMEM132A	2.165	****	1.321	*	1.842	***
EXO1	2.150	****	1.968	****	1.760	**
MYBL2	2.120	****	1.649	***	1.576	**
RRM2	2.013	***	2.001	****	1.805	**
CENPA	1.985	***	1.747	****	1.741	**
MELK	1.959	***	1.834	****	1.428	*
OR7E36P	1.949	***	1.397	*	1.445	*
SLC7A5	1.865	***	1.833	****	2.045	****
GINS1	1.851	***	1.668	***	1.461	*
MCM10	1.744	**	1.730	***	1.466	*
ORC6	1.667	**	1.394	*	1.549	*
CENPN	1.519	*	1.541	**	1.468	*

	10yr OS		10yr RFS		10yr DMFS	
**geneID**	HR_exp(coef)	*p* longrank	HR_exp(coef)	*p* longrank	HR_exp(coef)	*p* longrank
HJURP	2.373	****	2.057	****	1.328	0.101
MKI67	1.971	***	2.062	****	1.365	0.073
NCAPH	1.887	***	1.966	****	1.380	0.062
E2F8	1.702	**	1.304	0.064	1.759	**

	10yr OS		10yr RFS		10yr DMFS	
**geneID**	HR_exp(coef)	*p* longrank	HR_exp(coef)	*p* longrank	HR_exp(coef)	*p* longrank
HSPB1	1.560	*	1.168	0.277	0.898	0.532
RPP40	1.326	0.115	1.307	0.061	1.528	*
LRP8	1.240	0.229	1.553	**	1.215	0.261

	10yr OS		10yr RFS		10yr DMFS	
**geneID**	HR_exp(coef)	*p* longrank	HR_exp(coef)	*p* longrank	HR_exp(coef)	*p* longrank
PRR22	1.139	0.466	1.260	0.105	1.021	0.904
TUBA4A	1.125	0.510	1.171	0.269	1.231	0.229
IFI44L	0.941	0.733	1.205	0.191	1.210	0.268
STAT1	0.907	0.587	1.051	0.730	1.132	0.471
