# Supplementary material for: Cancer Grade Model: a multi-gene machine learning-based risk classification for improving prognosis in breast cancer
Source: Br J Cancer. 2021 Jun 15;125(5):748–58. doi: 10.1038/s41416-021-01455-1 (PMC8405688; doi:10.1038/s41416-021-01455-1)
Supplement: Supplementary file 6 — Supplementary Table S6 [file 41416_2021_1455_MOESM6_ESM.pdf]

Table S6: Survival analysis for genes identified as under-expressed in high-risk group (with low Hazard Ratio values)

Genes affecting Metastasis, Relapse, and Overall survival

geneID	10yr OS HR_exp(coef)	10yr OS *p* longrank	10yr RFS HR_exp(coef)	10yr RFS *p* longrank	10yr DMFS HR_exp(coef)	10yr DMFS *p* longrank
CX3CR1	0.385	****	0.583	***	0.653	*
ERBB4	0.445	****	0.539	****	0.468	****
DNAJC12	0.448	****	0.544	****	0.508	***
NAT1	0.461	****	0.615	***	0.500	***
PGR	0.462	****	0.627	**	0.359	****
ABAT	0.462	****	0.592	***	0.467	****
LINC00472	0.479	***	0.666	**	0.486	***
IL6ST	0.483	***	0.576	***	0.483	***
SCUBE2	0.508	***	0.704	*	0.545	***
ADRA2A	0.533	***	0.621	**	0.680	*
STC2	0.534	***	0.652	**	0.694	*
TBC1D9	0.542	***	0.664	**	0.492	****
BBOF1	0.570	**	0.501	****	0.692	*
TMC5	0.595	**	0.623	**	0.672	*
OSBPL1A	0.605	**	0.602	***	0.605	**
LAMP5	0.641	*	0.706	*	0.629	**
NME5	0.645	*	0.556	****	0.622	**
KIF13B	0.672	*	0.609	***	0.571	**

geneID	10yr OS HR_exp(coef)	10yr OS *p* longrank	10yr RFS HR_exp(coef)	10yr RFS *p* longrank	10yr DMFS HR_exp(coef)	10yr DMFS *p* longrank
SLC25A12	0.498	***	0.600	***	0.831	0.286
STARD13	0.535	***	0.623	**	0.724	0.064
WDR19	0.578	**	0.668	**	0.736	0.077
PSD3	0.606	**	0.652	**	0.895	0.525
ESD	0.606	**	0.611	***	0.903	0.556
CACNA1D	0.608	**	0.593	***	0.896	0.530
CIRBP	0.642	*	0.578	***	0.736	0.076
MATN3	0.687	*	0.625	**	0.764	0.138

geneID	10yr OS HR_exp(coef)	10yr OS *p* longrank	10yr RFS HR_exp(coef)	10yr RFS *p* longrank	10yr DMFS HR_exp(coef)	10yr DMFS *p* longrank
NAV2	0.668	*	0.768	0.066	0.884	0.473
FMO5	0.714	0.075	0.673	**	0.884	0.491
PDZRN3	0.734	0.084	0.669	**	0.942	0.729
DRC3	0.825	0.288	0.688	*	0.803	0.215
GLRB	0.743	0.115	0.778	0.095	0.671	*

geneID	10yr OS HR_exp(coef)	10yr OS *p* longrank	10yr RFS HR_exp(coef)	10yr RFS *p* longrank	10yr DMFS HR_exp(coef)	10yr DMFS *p* longrank
STK32B	0.733	0.100	0.839	0.229	0.738	0.100
PTPRT	0.778	0.177	0.938	0.656	0.793	0.209
NTRK2	0.800	0.222	0.819	0.179	0.814	0.248
PCSK6	0.834	0.314	0.788	0.098	0.729	0.075
CLMN	1.175	0.366	0.950	0.719	1.462	0.029
