# Supplementary material for: Cancer Grade Model: a multi-gene machine learning-based risk classification for improving prognosis in breast cancer
Source: Br J Cancer. 2021 Jun 15;125(5):748–58. doi: 10.1038/s41416-021-01455-1 (PMC8405688; doi:10.1038/s41416-021-01455-1)
Supplement: Supplementary file 7 — Supplementary Table S7 [file 41416_2021_1455_MOESM7_ESM.pdf]

Table S7: Reported gene biomarkers by other prognostic tests and CGM

EndoPredict	OncotypeDX	GGI			CGM	
BIRC5	BIRC5	BIRC5	DDX39A	RACGAP1	BIRC5	ERBB4*
UBE2C	MKI67	UBE2C	BBS1	CDK2	MKI67	HJURP*
L6ST	MYBL2	MKI67	FRY	UBE2S	MYBL2	KIF13B*
STC2	PGR	MYBL2	MCM2	SHMT2	UBE2C	PCSK6*
RBBP8	AURKA	AURKB	CDCA8	CDCA3	PGR	CACNA1D*
AZGP1	SCUBE2	CENPA	CCNE2	H2AFZ	AURKA	SLC25A12*
DHCR7	CCNB1	NCAPH	BLM	NDC80	SCUBE2	LRP8*
MGP	MMP11	MELK	BUB1	SIRT3	L6ST	OSBPL1A*
CALM2	CTSV	STARD13	BUB1B	KIFC1	STC2	TMC5*
OAZ1	GRB7	CENPN	CEP55	MAD2L1	AURKB	ORC6*
RPL37A	ERBB2	CDC20	IFT46	ORMDL2	CENPA	CIRBP*
	ESR1	CX3CR1	CCT5	C20orf24	NCAPH	STAT1*
	BCL2	TPX2	HMMR	KPNA2	MELK	ESD*
	GSTM1	WDR19	MCM4	KIF14	STARD13	PRR22*
	CD68	EXO1	CDC25A	PLK1	CENPN	CLMN*
	BAG1	TRIP13	CDKN3	CENPE	CDC20	PDZRN3*
	ACTB	PTTG1	CENPF	NUDT1	CX3CR1	TUBA4A*
	GAPDH	SLC7A5	CENPU	LAMB2	TPX2	DNAJC12*
	RPLP0	KIF2C	KIF11	LMNB1	WDR19	IFI44L*
	GUSB	MCM10	TTK	TUBA1C	EXO1	TBC1D9*
	TFRC	CCNB2	ESPL1	UBE2N	TRIP13	HSPB1*
		RRM2	FEN1	SESN1	PTTG1	TMEM132A*
		CMC2	FOXM1	IFT88	SLC7A5	PSD3*
		NCAPG	FAM64A	CYBRD1	KIF2C	NTRK2*
		OIP5	KIF4A	SPAG5	MCM10	OR7E36P*
		RNASEH2A	TIMELESS	POLQ	RRM2	GLRB*
		AURKB	PIGV	GMPS	CCNB2	ABAT*
		A7URKA	PARPBP	DLGAP5	NME5*	MATN3*
		NUSAP1	KIF20A	TROAP	PTPRT*	ADRA2A*
		CCNA2	C11orf63		LINC00472*	ADGRG1*
		PRC1	KIF15		STK32B*	NAV2*
		ASPM	TPT1		BBOF1*	FMO5*
		CCNB1	MARS		E2F8*	GINS1*
		DONSON	HMGB3		DRC3*	RPP40*
		GTSE1	ZWINT		NAT1*	LAMP5*

* CGM's uniqe genes
