# Supplementary material for: Cancer Grade Model: a multi-gene machine learning-based risk classification for improving prognosis in breast cancer
Source: Br J Cancer. 2021 Jun 15;125(5):748–58. doi: 10.1038/s41416-021-01455-1 (PMC8405688; doi:10.1038/s41416-021-01455-1)
Supplement: Supplementary file 8 — Supplementary Table S8 [file 41416_2021_1455_MOESM8_ESM.pdf]

Table S8: Pairwise comparison of CGM with other prognostic test results

EndoPredict_CGM				
	low_risk_EndoPredict	high_risk_EndoPredict	total	%
low_risk_CGM	379	751	**1130**	**37.31**
high_risk_CGM	16	1883	**1899**	**62.69**
**total**	**395**	**2634**	**3029**	
**%**	**13.04**	**86.96**		**0.74**
**OncotypeDx*_CGM**				
	low_risk_Oncotypedx	high_risk_Oncotypedx	total	%
low_risk_CGM	567	94	**661**	**29.61**
high_risk_CGM	101	1470	**1571**	**70.39**
**total**	**668**	**1564**	**2232**	
**%**	**29.93**	**70.07**		**0.91**
**CGM_GGI****				
	high_risk_CGM	high_risk_CGM		
low_risk_GGI	527	308	**835**	**59.26**
high_risk_GGI	23	551	**574**	**40.74**
**total**	**550**	**859**	**1409**	
**%**	**39.03**	**60.97**		**0.76**
**OncotypeDx_GGI**				
	low_risk_Oncotypedx	high_risk_Oncotypedx	total	%
low_risk_GGI	288	176	**464**	**47.11**
high_risk_GGI	27	494	**521**	**52.89**
**total**	**315**	**670**	**985**	
**%**	**31.98**	**68.02**		**0.79**
**OncotypeDx_EndoPredict**				
	low_risk_EndoPredict	high_risk_EndoPredict	total	%
low_risk_Oncotypedx	296	372	**668**	**29.93**
high_risk_Oncotypedx	4	1560	**1564**	**70.07**
**total**	**300**	**1932**	**2232**	
**%**	**13.44**	**86.56**		**0.83**
**EndoPredict_GGI**				
	low_risk_EndoPredict	high_risk_EndoPredict	total	%
low_risk_GGI	180	655	**835**	**59.26**
high_risk_GGI	3	571	**574**	**40.74**
**total**	**183**	**1226**	**1409**	
**%**	**12.99**	**87.01**		**0.53**

* 797 samples are predicted as medium-risk group, so they are removed form the comparison

** 1620 samples do not have grade information, therefore GGI cannot be applied on them
