# Supplementary material for: Cancer Grade Model: a multi-gene machine learning-based risk classification for improving prognosis in breast cancer
Source: Br J Cancer. 2021 Jun 15;125(5):748–58. doi: 10.1038/s41416-021-01455-1 (PMC8405688; doi:10.1038/s41416-021-01455-1)
Supplement: Supplementary file 9 — Supplementary Figure Legends [file 41416_2021_1455_MOESM9_ESM.docx]

**Supplementary figure legends**

**Figure S1:** Data selection workflow. All Affymetrix Human Genome breast cancer datasets with information about Histological grade/Overall survival/Relapse-free survival/distant metastasis-free survival were obtained from GEO Database. Datasets with less than 25 samples, published before 2006, and samples with prior treatment were excluded. 33 datasets were selected that contain 5031 tumour samples. The final integrated dataset was split to development-dataset (grade-1 and grade-3 samples) and prediction-dataset (grade-2 and unknown grade samples) for building and evaluating the proposed model, respectively

**Figure S2:** Selection of the 70 top genes with the highest Gain value. Performance of the method according to the number of top selected genes.

**Figure S3:** (a) Survival analysis based for all samples according to histological grade. (b) Hierarchical clustering of the development-dataset (grade-1 and grade-3 samples) on 70 selected genes. (c) SHAP-based analysis for the development-dataset. For each point, the colour indicates the value of the corresponding feature in the sample and its vertical position reflects the impact in prediction.

**Figure S4:** (a) Association between CGM, tumour stage, and ER status with relapse-free survival through multivariable analysis, (b) multivariable analysis of the prognostic methods (GGI, OncotypeDX, and EndoPredict) based on time to relapse, (c) KM plot of the joint distribution of stage, ER status and CGM based on RFS.

**Figure S5:** Heatmap of gene expression of normal samples (yellow), low-risk (blue) and high-risk (pink) tumours.

**Figure S6:** Reactome pathway enrichment analysis on genes linked to high risk disease. (a) Pathways linked to genes found to be overexpressed in high risk and (b) Pathways for genes that were under-expressed in the high-risk cohort. (c) Illustration of the most affected pathways and related genes in the high-risk group.

**Figure S7:** Kaplan-Meier plots for distant metastasis-free survival. KM-plot of patients in the prediction-dataset (grade-2 and unknown grade) based on high- (pink) or low-risk (blue) groups, categorised by GGI, OncotypeDX, Endopredict, CGM (shown clockwise from top left).
