# Supplementary figures and images for: Cancer Grade Model: a multi-gene machine learning-based risk classification for improving prognosis in breast cancer
Source: Br J Cancer. 2021 Jun 15;125(5):748–58. doi: 10.1038/s41416-021-01455-1 (PMC8405688; doi:10.1038/s41416-021-01455-1)

**Figure S1**

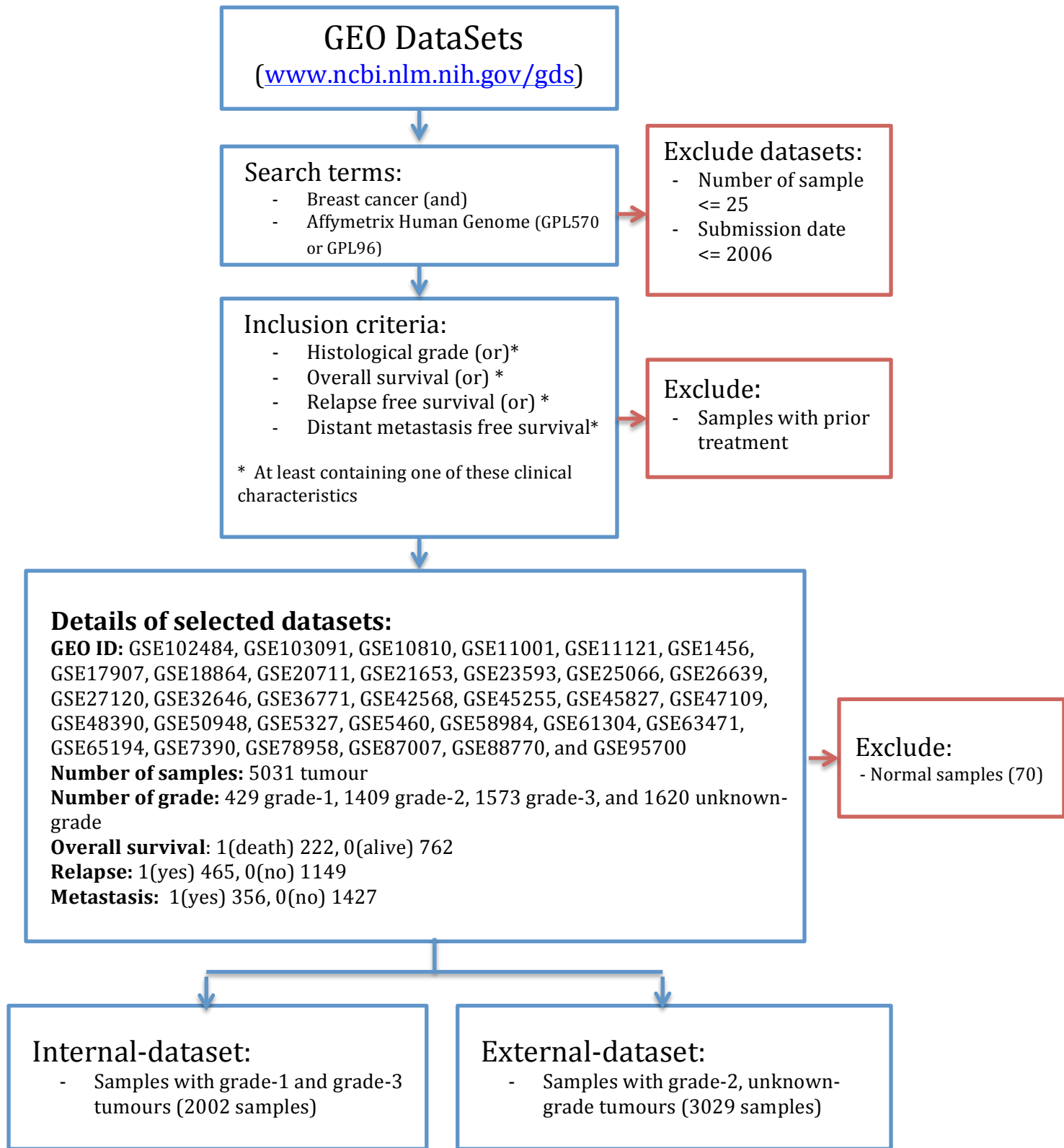

Supplement: Supplementary file 10 — Supplementary Figure S1 [file 41416_2021_1455_MOESM10_ESM.pdf]

Supplementary Figure S2

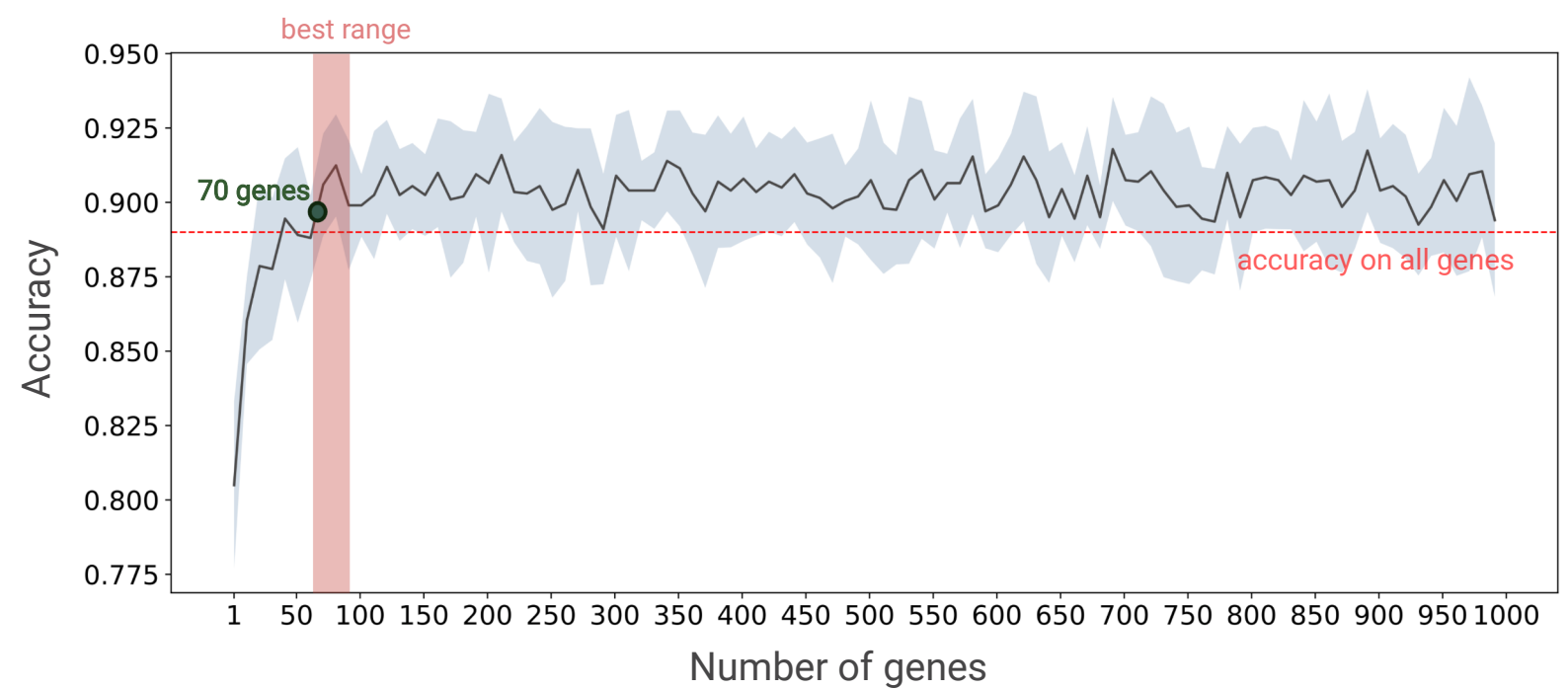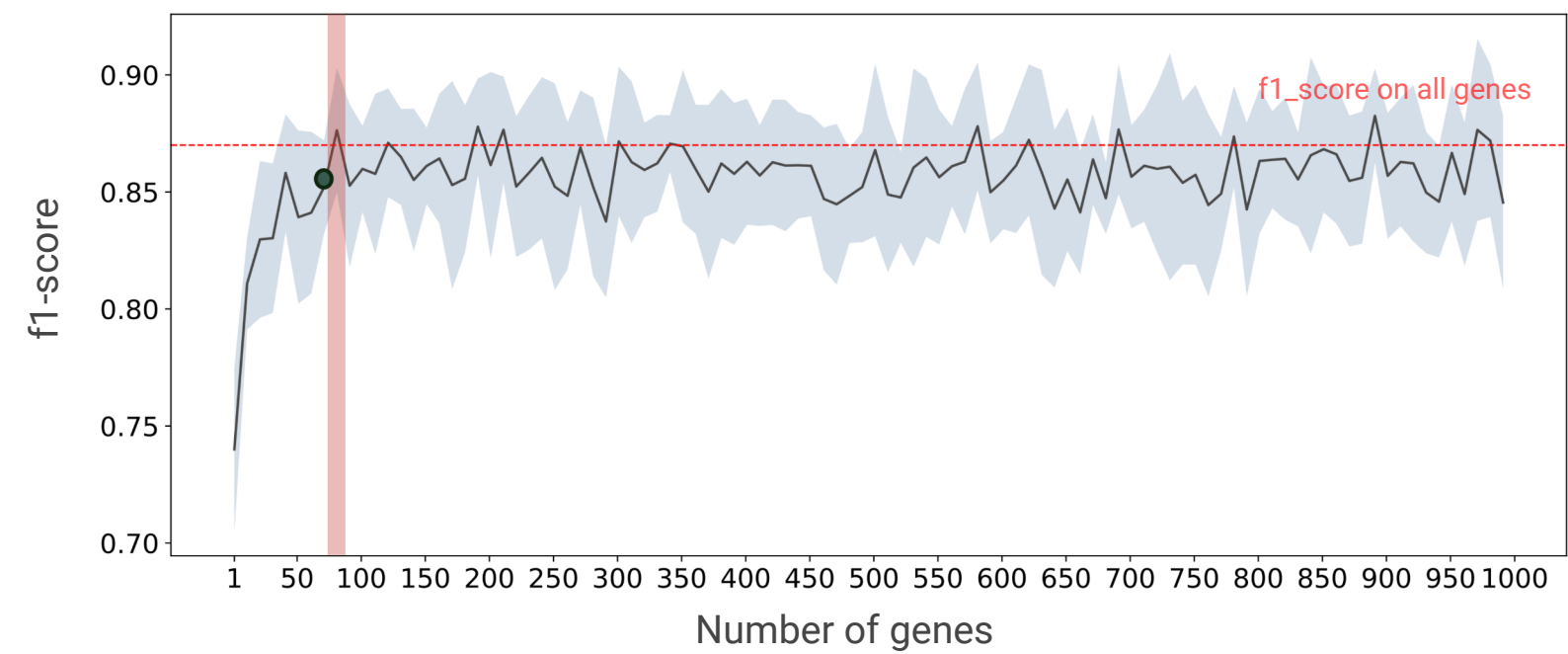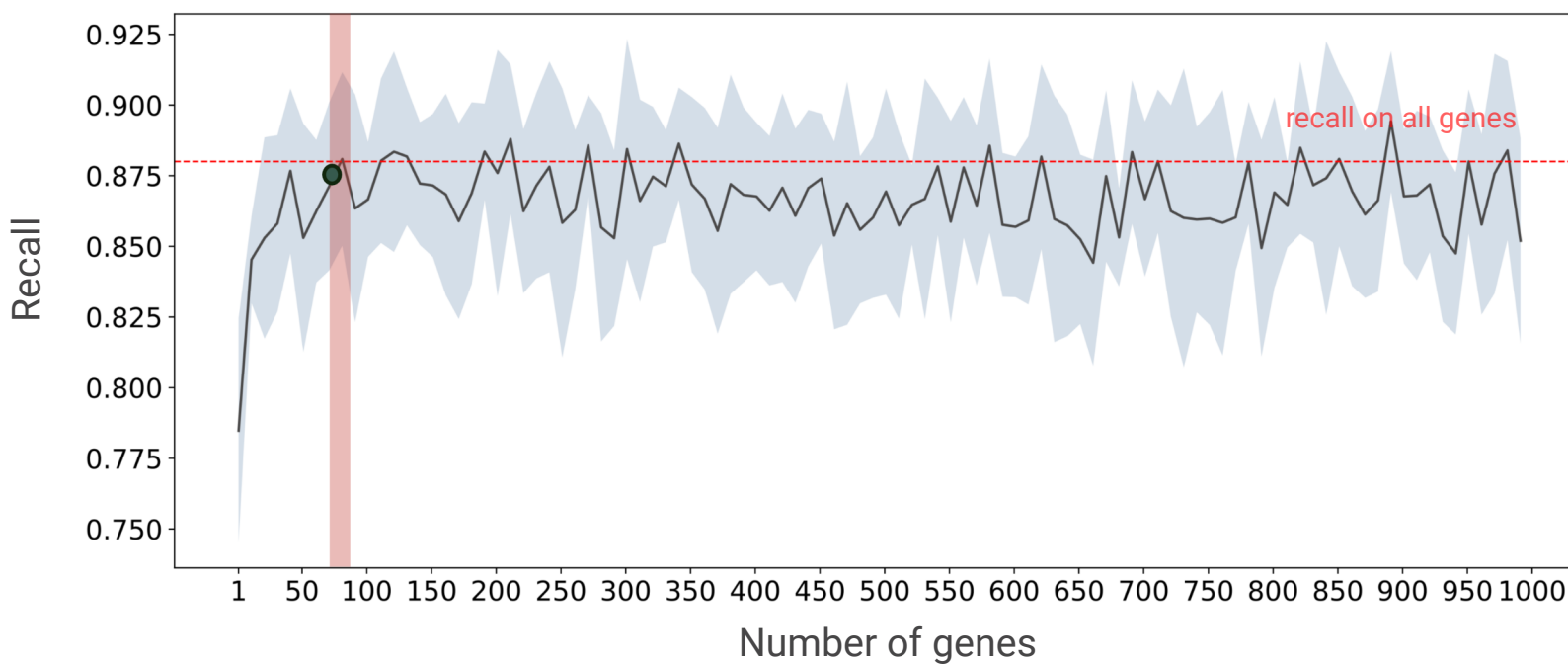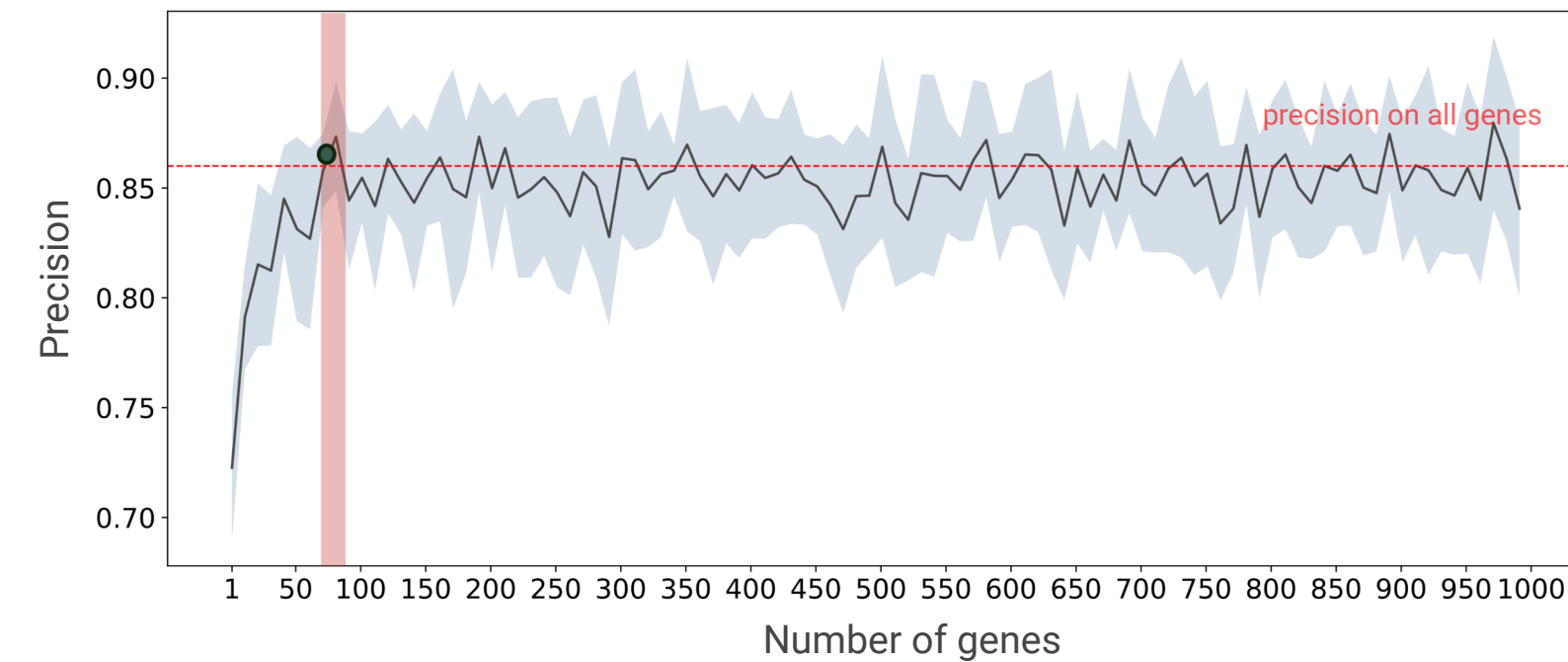

Supplement: Supplementary file 11 — Supplementary Figure S2 [file 41416_2021_1455_MOESM11_ESM.pdf]

Supplementary Figure S3

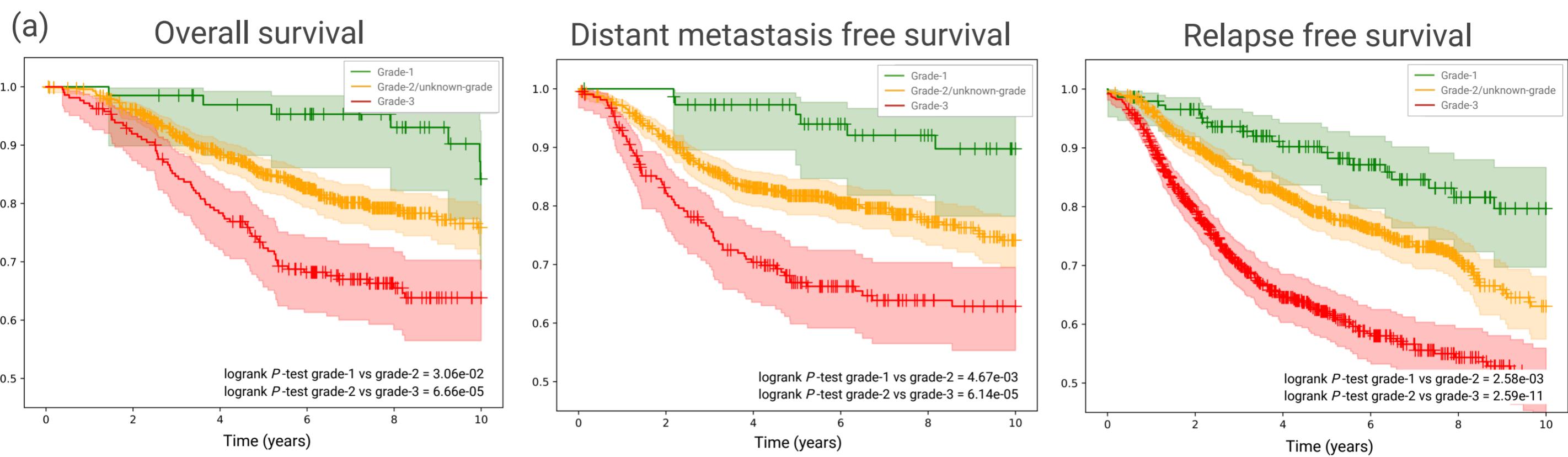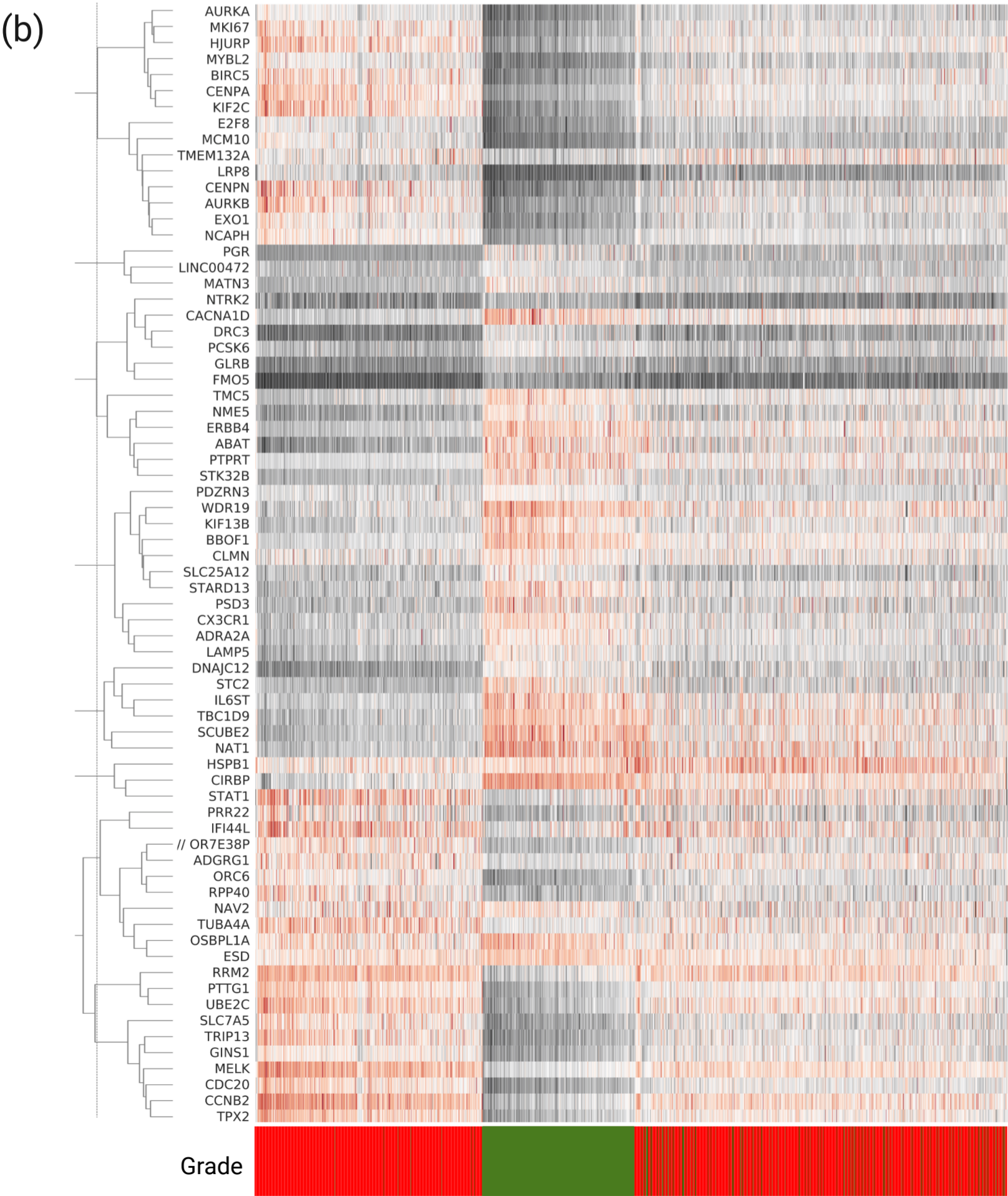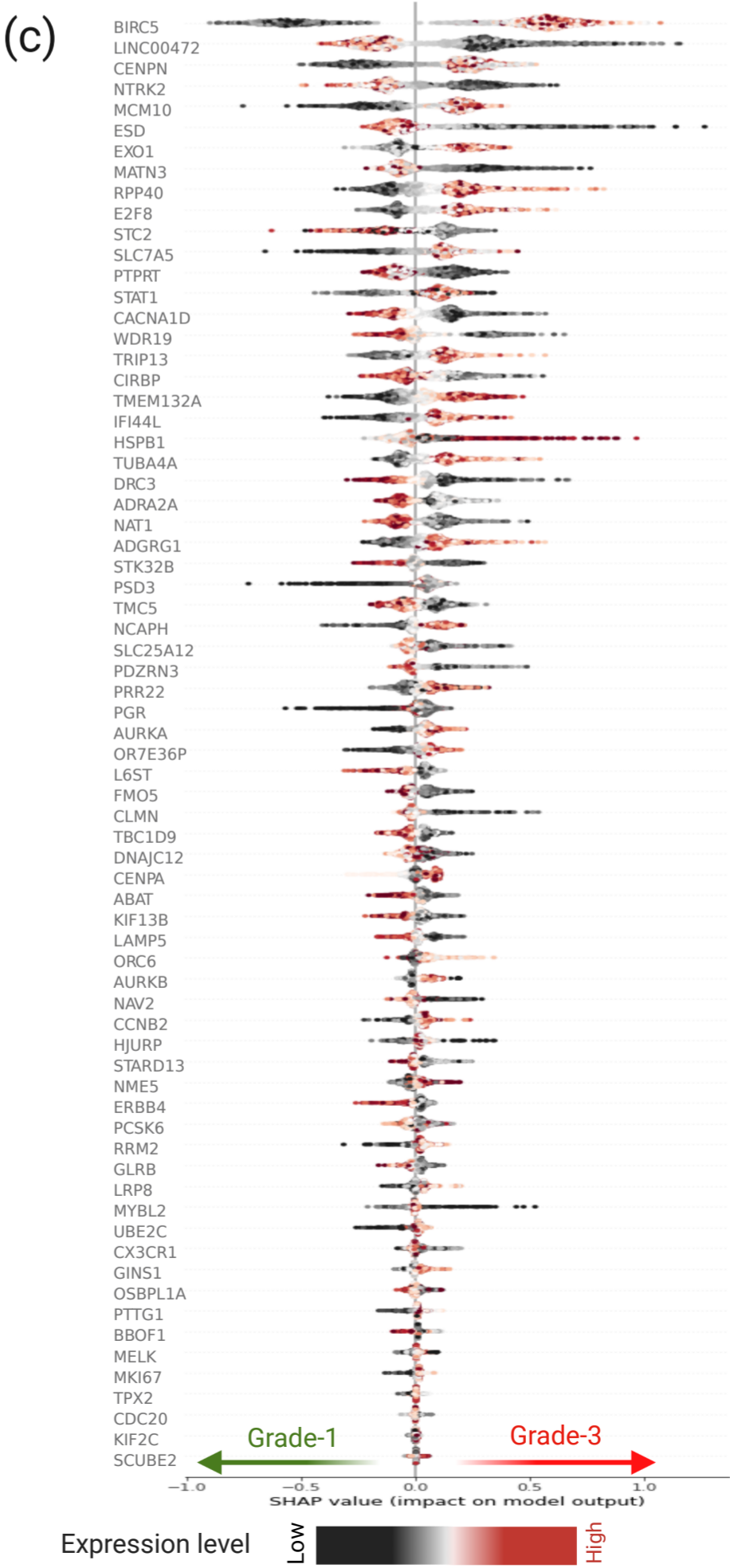

Supplement: Supplementary file 12 — Supplementary Figure S3 [file 41416_2021_1455_MOESM12_ESM.pdf]

Supplementary Figure S4

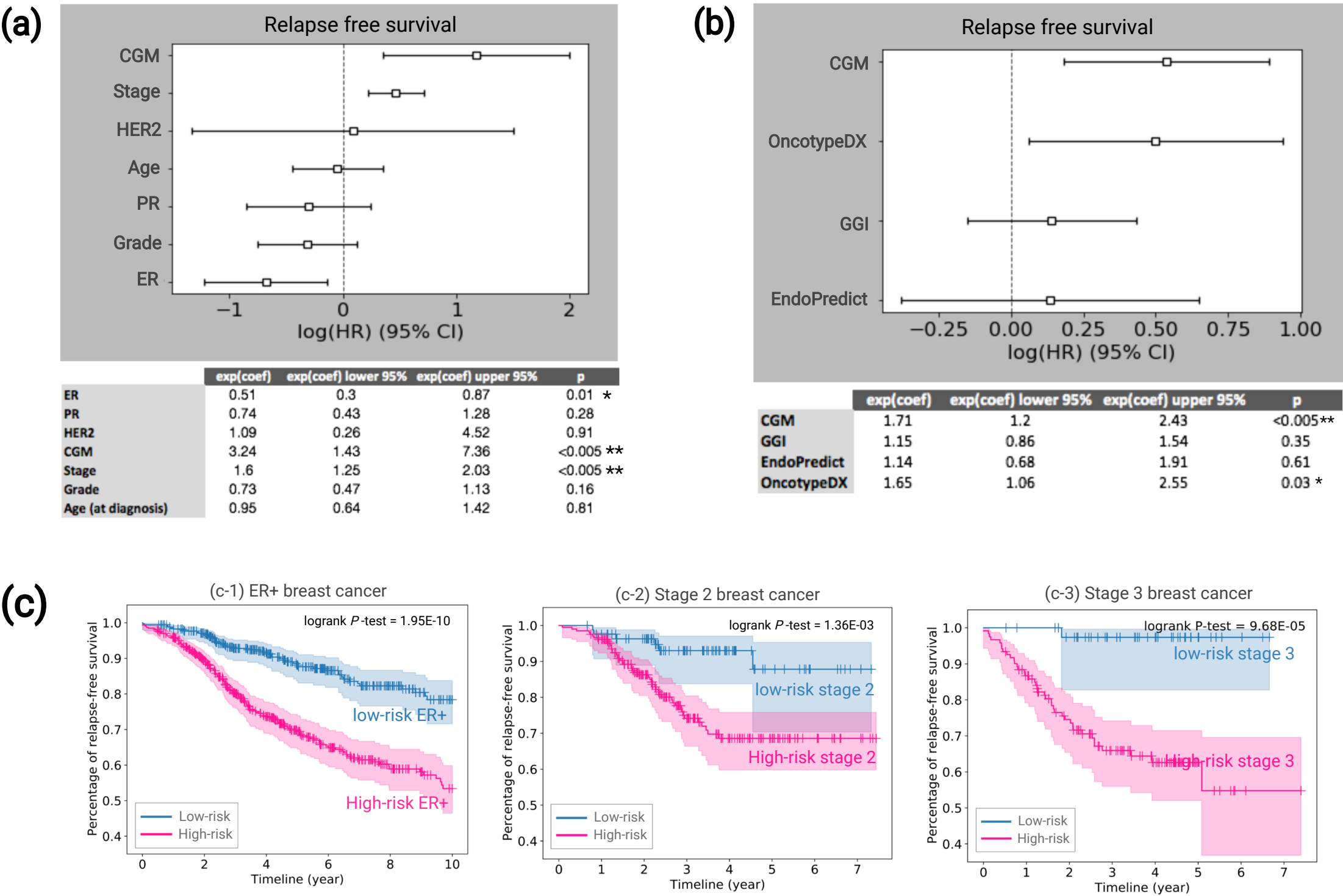

Supplement: Supplementary file 13 — Supplementary Figure S4 [file 41416_2021_1455_MOESM13_ESM.pdf]

Supplementary Figure S5

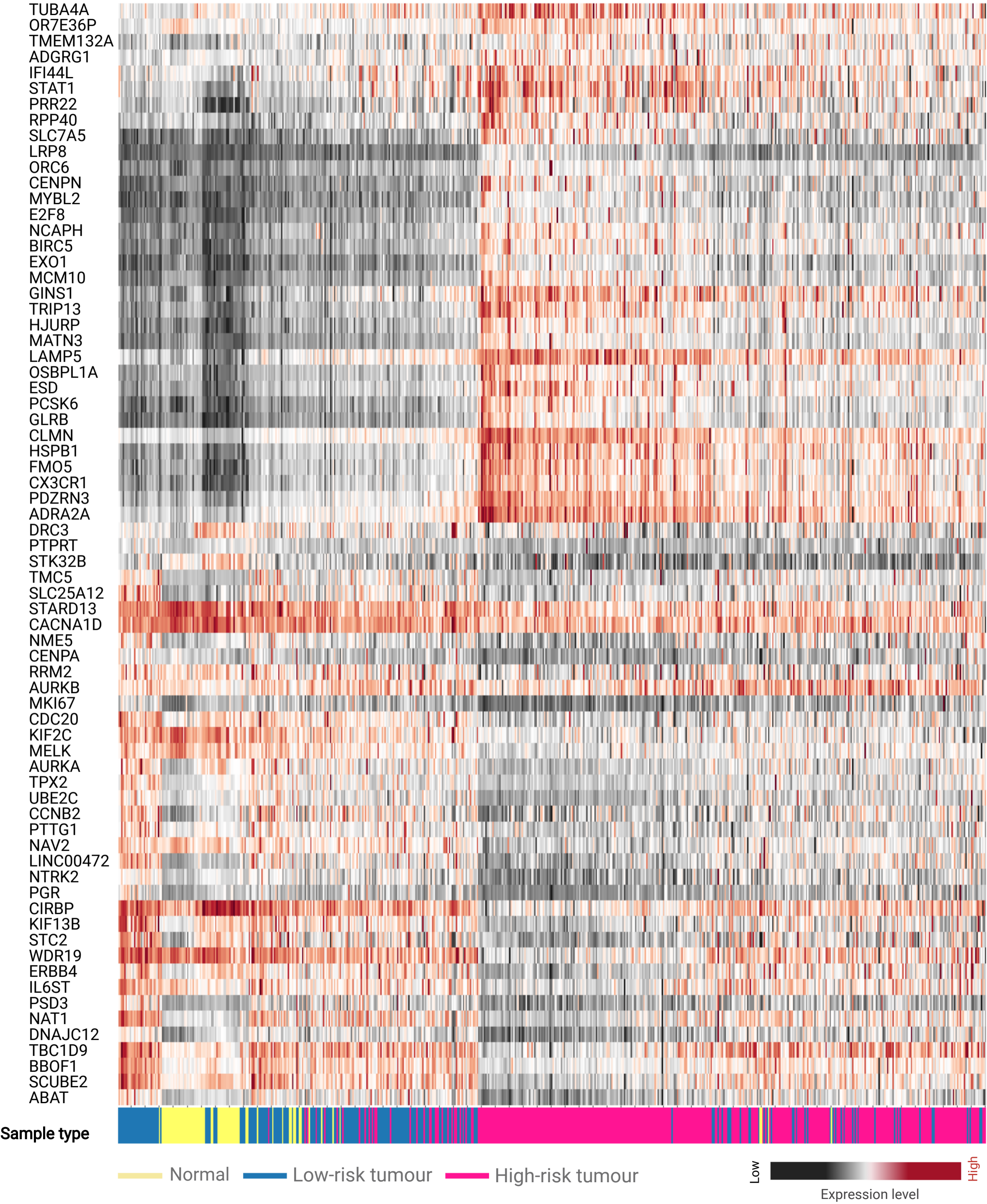

Supplement: Supplementary file 14 — Supplementary Figure S5 [file 41416_2021_1455_MOESM14_ESM.pdf]

Supplementary Figure S7

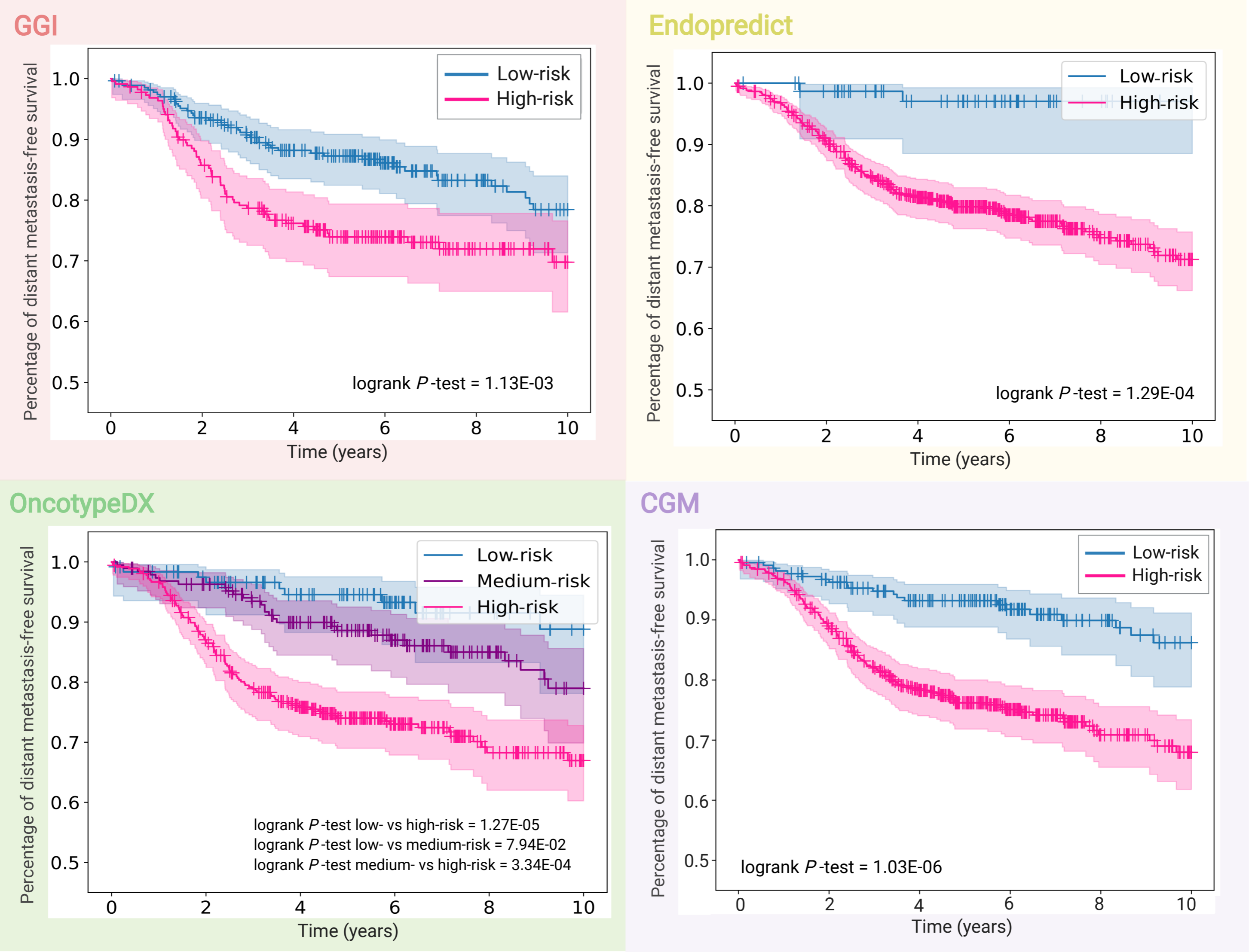

Supplement: Supplementary file 16 — Supplementary Figure S7 [file 41416_2021_1455_MOESM16_ESM.pdf]
