# Supplementary material for: Cancer Grade Model: a multi-gene machine learning-based risk classification for improving prognosis in breast cancer
Source: Br J Cancer. 2021 Jun 15;125(5):748–58. doi: 10.1038/s41416-021-01455-1 (PMC8405688; doi:10.1038/s41416-021-01455-1)

Supplementary Figure S6

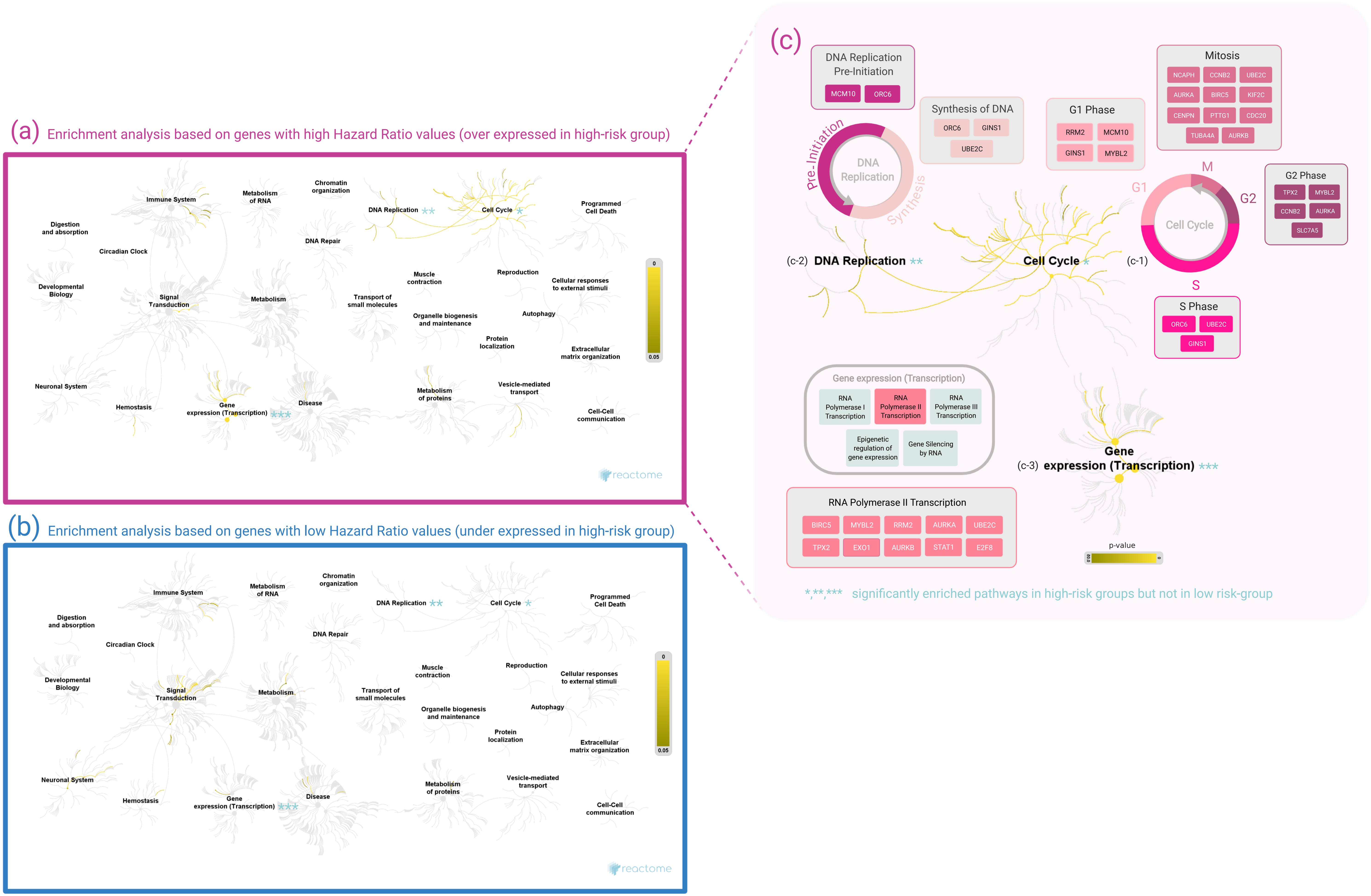

Supplement: Supplementary file 15 — Supplementary Figure S6 [file 41416_2021_1455_MOESM15_ESM.pdf]
